# Smooth Muscle Enriched Long Noncoding RNA (*SMILR*) Regulates Cell Proliferation

**DOI:** 10.1161/CIRCULATIONAHA.115.021019

**Published:** 2016-05-23

**Authors:** Margaret D. Ballantyne, Karine Pinel, Rachel Dakin, Alex T. Vesey, Louise Diver, Ruth Mackenzie, Raquel Garcia, Paul Welsh, Naveed Sattar, Graham Hamilton, Nikhil Joshi, Marc R. Dweck, Joseph M. Miano, Martin W. McBride, David E. Newby, Robert A. McDonald, Andrew H. Baker

**Affiliations:** From BHF Glasgow Cardiovascular Research Centre, University of Glasgow, United Kingdom (M.D.B., R.D., L.D., R.M., R.G., P.W., N.S., M.W.N., R.A.M., A.H.B.); British Heart Foundation/University of Edinburgh Centre for Cardiovascular Science, Edinburgh, United Kingdom (M.D.B., K.P., A.T.V., N.J., M.R.D., D.E.N., R.A.M., A.H.B.); Glasgow Polyomics, College of Medical, Veterinary and Life Sciences, The University of Glasgow, United Kingdom (G.H.); and Aab Cardiovascular Research Institute, University of Rochester School of Medicine and Dentistry, NY (J.M.M.).

**Keywords:** atherosclerosis, cell proliferation, microRNAs, RNA, untranslated, plasma protein, human

## Abstract

**Background—:**

Phenotypic switching of vascular smooth muscle cells from a contractile to a synthetic state is implicated in diverse vascular pathologies, including atherogenesis, plaque stabilization, and neointimal hyperplasia. However, very little is known about the role of long noncoding RNA (lncRNA) during this process. Here, we investigated a role for lncRNAs in vascular smooth muscle cell biology and pathology.

**Methods and Results—:**

Using RNA sequencing, we identified >300 lncRNAs whose expression was altered in human saphenous vein vascular smooth muscle cells following stimulation with interleukin-1α and platelet-derived growth factor. We focused on a novel lncRNA (Ensembl: RP11-94A24.1), which we termed smooth muscle–induced lncRNA enhances replication (*SMILR*). Following stimulation, *SMILR* expression was increased in both the nucleus and cytoplasm, and was detected in conditioned media. Furthermore, knockdown of *SMILR* markedly reduced cell proliferation. Mechanistically, we noted that expression of genes proximal to *SMILR* was also altered by interleukin-1α/platelet-derived growth factor treatment, and *HAS2* expression was reduced by *SMILR* knockdown. In human samples, we observed increased expression of *SMILR* in unstable atherosclerotic plaques and detected increased levels in plasma from patients with high plasma C-reactive protein.

**Conclusions—:**

These results identify *SMILR* as a driver of vascular smooth muscle cell proliferation and suggest that modulation of *SMILR* may be a novel therapeutic strategy to reduce vascular pathologies.

## Abstract 2

Supplemental Digital Content is available in the text.

Vessel wall remodeling is an integral pathological process central to cardiovascular diseases including atherogenesis, plaque rupture, and neointimal hyperplasia–associated vein graft failure and in-stent restenosis.^1,2^ Resident vascular smooth muscle cells (VSMCs) are typically quiescent and contractile in the normal physiological state. However, following pathological or iatrogenic vascular injury, the release of cytokines and growth factors from VSMCs, aggregated platelets, and inflammatory cells on the damaged intimal surface leads to phenotypic switching of VSMCs and the adoption of a more synthetic, pro-proliferative, and promigratory state.^3^ In the setting of the pathological injury of atherosclerosis, VSMCs not only contribute to the atherogenic process itself but can also engender plaque stabilization through the generation of a thick-capped fibroatheroma. For acute iatrogenic vascular injury, overexuberant proliferation of VSMC subpopulations promotes neointimal hyperplasia leading to luminal narrowing such as seen in vein graft failure or in-stent restenosis.^4^ Phenotypic switching of VSMCs and release of cytokines and growth factors are therefore critical in vascular disease, and understanding the mechanisms involved is critical to gain insights into the pathology and identify new opportunities for therapies.

**Clinical Perspective on p 2065**

The highly conserved interleukin-1α (IL1α) and platelet-derived growth factor (PDGF) pathways play prominent roles in VSMC-associated pathologies.^1,5^ IL1α is a central mediator in the cytokine cascade and a potent activator of vascular cytokine production. Furthermore, previous studies have demonstrated that ligation injury results in reduced neointimal formation in IL1 receptor knockout mice.^6^ Downstream mediators include the signaling molecules MEKK1 and p38 and the transcription factor NF-κB that activate mediators of inflammation and cellular migration.^7^ PDGF is a potent mitogen and chemoattractant, and expression is increased following vascular injury.^8^ Conversely, a reduction in PDGF expression reduces intimal thickening and cellular content of the neointima.^9^ Activation of both IL1α and PDGF signaling pathways simultaneously can activate common downstream targets leading to additive or synergistic effects. This includes activation of NF-κB leading to the upregulation of metalloproteinase 3 and 9,^10^ genes critical in the development of vasculoproliferative pathologies.

Over the past decade, there has been substantial interest in determining the complex interactions between hierarchical levels of gene regulation. Up to 90% of the human genome is transcribed at different developmental stages and only ≈2% of RNA molecules are translated into protein.^11^ The functional complexity of organisms therefore appears to be reliant on noncoding RNA molecules. Noncoding RNAs are subdivided into several classes, including microRNA (miRNA) and long noncoding RNA (lncRNA). miRNAs are abundantly expressed in vascular tissues and play an important role in vascular pathology. Interestingly, recent studies have demonstrated that miRNAs are capable of being released into the blood from injured cells. These miRNAs are relatively stable and have been reported as biomarkers for several disease states, including myocardial infarction^12^ and heart failure.^13,14^ Although the role of miRNAs is reasonably established in the setting of cardiovascular pathology, relatively little is known about the role of lncRNAs. lncRNAs are capable of regulating target DNA, RNA, and protein at the pre- and posttranscriptional level. It is becoming clear that lncRNAs play a pivotal role in cellular physiology and pathology via localization in subpopulations of cells and through highly controlled temporal expression.^15^ However, detailed insights into their regulation and biological roles are only beginning to emerge. In the vascular setting, *SENCR* and *MALAT1* have been implicated in the control of vascular cell migration and endothelial cell sprouting, respectively.^16,17^ Interestingly, *SENCR* is implicated in phenotypic switching of VSMCs to a more promigratory phenotype because knockdown of this lncRNA downregulates contractile genes.^17^ A greater understanding of lncRNAs in quiescent and proliferative VSMCs may provide valuable insight into the specific roles of lncRNAs in response to pathological processes.

## Methods

### Human Tissue Samples

Surplus human saphenous vein tissue was obtained from patients undergoing coronary artery bypass grafting. Carotid plaques were obtained from patients undergoing endarterectomy following an acute and symptomatic neurovascular event. Human plasma samples were used from a previously published study: Carotid Ultrasound and Risk of Vascular disease in Europeans and South Asians (CURVES).^2^ All patients gave their written, informed consent. All procedures had local ethical approval (06/S0703/110, 12/WS/0227, 09/S0703/118, and 12/NW/0036). All studies were approved by East and West Scotland Research Ethics Committees, and all experiments were conducted according to the principles expressed in the Declaration of Helsinki.

### Tissue and Cell Culture

All cells were maintained at 37°C in a humidified atmosphere containing 5% CO_2_. Primary human saphenous vein–derived endothelial cells (HSVECs) were isolated by a modified version of the protocol described by Jaffe and colleagues^18^ and maintained in large-vessel endothelial cell culture medium supplemented with 20% fetal calf serum (Life Technologies, Paisley, UK). Primary human saphenous vein–derived smooth muscle cells (HSVSMCs) were isolated from medial explants^19^ and maintained in Smooth Muscle Cell Growth Medium 2 (PromoCell, Heidelberg, Germany) with supplements. Human coronary artery VSMCs were purchased from Lonza (Basel, Switzerland) and maintained in VSMC media as above.

### Sample Preparation for RNA-seq Library Construction and Analysis

HSVSMCs were plated, quiesced in medium containing 0.2% fetal calf serum for 48 hours before the stimulation with 10 ng/mL IL1α, 20 ng/mL PDGF (R&D Systems) or a combination of both for 72 hours. Total RNA was processed through miRNeasy kit (Qiagen, Hilden, Germany) following the manufacturer’s instructions, treated with DNase 1 (amplification grade; Sigma, St. Louis, MO) to eliminate genomic DNA contamination and quantified by using a NanoDrop ND-1000 Spectrophotometer (Nano-Drop Technologies, Wilmington, DE). Following bioanalyzer quality control for RNA integrity number values >8, RNA sequencing (RNA-seq) was performed on ribosomal-depleted RNA using an Illumina Hiseq platform by Beckman Coulter Genomics. Paired-end sequencing was performed with a read depth of 70 million (n=4/group). RNA-seq reads were processed and trimmed to ensure quality, adapter sequences were removed using Flexbar^20^ and mapped to the Ensembl annotation of GRCh37.75 using the TopHat2 version 2.0.9.^21^ The transcriptome was assembled from the aligned reads and quantified using Cufflinks version 2.2.1.^22^ The differential expression levels between the groups were assessed using Cuffdiff version 2.2.1.^23^ The data set is deposited in the Gene Expression Omnibus (GEO) repository, study number GSE69637. The biotype of each transcript was annotated according to the Ensembl database. Normalization and statistical analysis of differentially expressed transcripts were performed by using edgeR and data filtered to find transcripts that were differentially expressed (*P*<0.01) between 0.2% media and each treatment group. Differentially expressed lncRNAs, between control and both IL1α/PDGF treatments, were explored by using more stringent criteria (*P*<0.01, false discover rate [FDR] <0.01, log fold change>2) and filtered according to transcript abundance (fragments per kilobase of exon per million fragments mapped [FPKM]>1 in at least 1 group). Data outputs such as pie charts and heatmaps were generated using R. Ingenuity pathway analysis was performed by using protein-coding genes differentially expressed (FDR<0.01) from Edge R analysis.

### Assessment of RNA Secretion From HSVSMC

RNA extraction on conditioned HSVSMC media was performed by using a standard volume (2 mL). The conditioned media was first centrifuged (10 minutes; 2000*g*; 4°C) to remove all cells and debris. After addition of 1.4 mL of QIAzol (Qiagen), 3 µL of *Caenorhabditis elegans* total RNA at 25 ng/µL was added to each sample. Following 5-minute incubation at room temperature, 140 µL of chloroform was added and samples centrifuged (15 minutes; 15 000*g*; 4°C). The clear upper aqueous phase was used to isolate RNA by using the miRNEasy mini kit (Qiagen) as previously described with alteration of the final wash step (75% ethanol in diethylpyrocarbonate water). Different quantities of total RNA were spiked and a dose-response effect was observed (Figure IA in the online-only Data Supplement). The quality of the amplicon was assessed via analysis of melting curves (Figure IB in the online-only Data Supplement) and subsequent visualization on agarose gel (Figure IC in the online-only Data Supplement). This showed a unique amplification product corresponding to the cDNA fragment of *ama-1*. Because of the correlation observed between the quantity of spike-in and *ama-1* expression (Figure ID in the online-only Data Supplement), we used 75 ng in all subsequent extractions. This amount allowed reproducibility of our method, with the Ct values of *ama-1* being 29.4±0.3 across 5 separate extractions in nonconditioned media (Figure IE in the online-only Data Supplement).

### Gene Expression qRT-PCR

For gene expression analysis, cDNA for mRNA analysis was obtained from total RNA using the Multiscribe Reverse Transcriptase (Life Technologies, Paisley, UK). Quantitative real-time polymerase chain reaction (qRT-PCR) was performed using Power SYBR green (Life Technologies) with custom PCR primers (Eurofins MWG, Ebersberg, Germany), the specificity of these primers was confirmed by performing a melting curve and running their PCR produce on a gel (Table I in the online-only Data Supplement – primer sequences). Ubiquitin C was selected as housekeeping gene because of its stability across all groups studied. Fold changes were calculated by using the 2^-^ΔΔ^Ct^ method.^23^

### Statistical Analysis

Statistical analysis was performed according to figure legends. Data in graphs are shown on relative expression scales as referenced by Livak and Schmittgen.^24^ Data are given as both mean±standard deviation (shown as bars and whiskers) and also as the individual points to clearly represent the data. Note that as the relative expression scale is inherently skewed; the bars indicate the geometric mean of the relative expression fold change, and the standard deviation whiskers denote the relative expression fold change equivalent to an increase of 1 standard deviation above the mean on the log-transformed scale. All statistical analysis is performed on the dCt scale (a logarithmic transformation of the data shown on the relative quantification in the plots).^24^ No evidence of unequal variances across groups was found for any of analyses of the dCt scale data using the Levene test on Minitab version 17 before statistical analysis. Comparisons between 2 groups were analyzed using 2-tailed unpaired or paired Student *t* test. One-way analysis of variance with Tukey post hoc or 1-way analysis of variance multiple-comparisons test for pooled samples, via Graph Pad Prism version 5.0, was used for comparisons among ≥3 groups. Statistical significance is denoted by a *P* value of <0.05.

## Results

### Induction of Inflammatory and Cell Cycle Pathways by IL1α and PDGF

**Figure 1.**
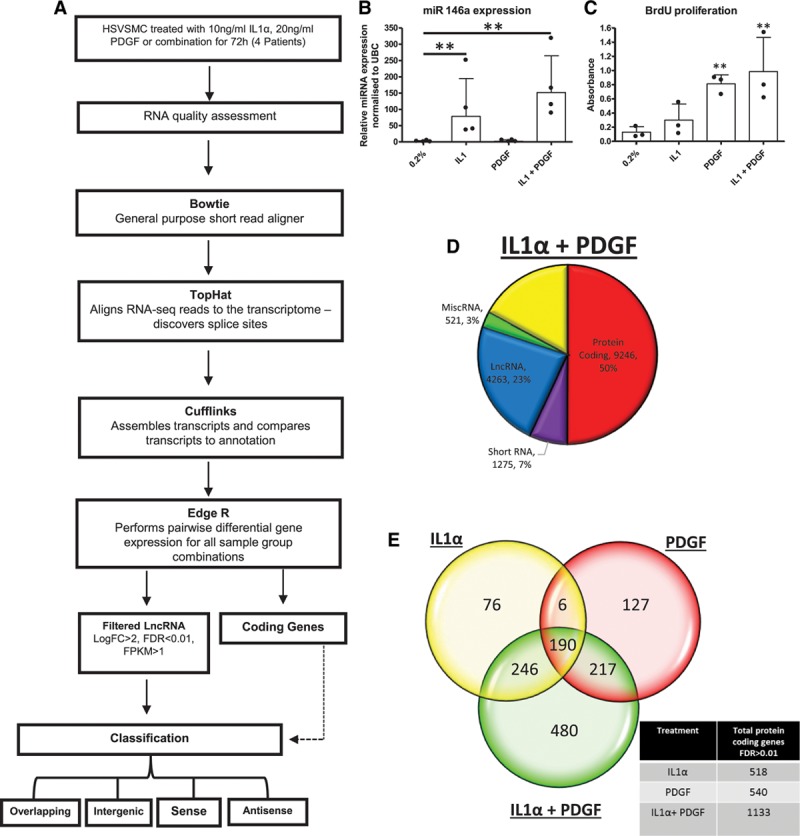
RNA sequencing shows IL1α and PDGF induction of inflammatory and cell cycle pathways. **A**, Study design for determination of the transcriptome in quiescent and stimulated HSVSMCs. HSVSMCs were treated for 72 hours, RNA quality was assessed and subjected to RNA-seq following the Tuxedo pipeline for analysis. **B**, Known inflammatory microRNA, miR146a, is upregulated by IL1α (n=4). **P<0.01 vs 0.2% condition. Multiple comparison 1-way ANOVA. **C**, BrdU incorporation as an indirect marker of proliferation was assessed in all patients (n=3). **P<0.01 vs 0.2% condition. **D**, Biotype distribution of all transcripts identified by RNA-seq analysis generated from HSVSMCs treated with IL1α and PDGF, cutoff at FPKM>0.1 **E**, Venn diagram indicating overlap of protein-coding genes with altered expression (analyzed using EdgeR, FDR<0.01) across each treatment. ANOVA indicates analysis of variance; BrdU, bromodeoxyuridine; FC, fold change; FDR, false discovery rate; FPKM, fragments per kilobase of exon per million fragments mapped; HSVSMC, human saphenous vein–derived smooth muscle cell; IL1α, interleukin-1α; lncRNA, long noncoding RNA; miR, microRNA; miscRNA, miscellaneous RNA; miRNA, microRNA; PDGF, platelet-derived growth factor; and UBC, ubiquitin C.

We sought to identify lncRNAs that are regulated during the induction of proliferative and inflammatory pathways in HSVSMCs. RNAs were identified by using RNA-seq of HSVSMC treated for 72 hours (Figure 1A). Activation of the IL1α and PDGF signaling pathways was confirmed by the presence of the inflammatory miRNA miR-146a (Figure1B) and induction of VSMC proliferation (Figure 1C). The RNA sequencing obtained an average of 70 million reads per sample, with 93.5% aligning to the GRCh37 genome reference files. The majority of reads, under all conditions, corresponded to mRNA (49.6±0.48%; Figure 1D and Figure IIA in the online-only Data Supplement). To identify the biological function, networks, and canonical pathways that were affected by VSMC stimulation, we performed ingenuity pathway analysis after RNA-seq analysis. Ingenuity pathway analysis confirmed that the mRNAs with altered expression following IL1α treatment were significantly enriched in pathways related to cellular movement and inflammatory disease (Table II in the online-only Data Supplement), whereas PDGF stimulation led to the marked enrichment in cell cycle pathways (Table III in the online-only Data Supplement). Interestingly, costimulation led to enrichment in cell cycle and cardiovascular development pathways (Table IV in the online-only Data Supplement). Further analysis of differentially expressed mRNAs with a stringent cutoff of FDR<0.01 identified 518 protein-coding genes altered following IL1α treatment and 540 following PDGF treatment. Notably, dual stimulation altered 1133 known protein-coding genes with 480 uniquely associated with dual stimulation and not affected by IL1α or PDGF treatment alone (Figure 1E and Figure IIB in the online-only Data Supplement).

### Identification of Differentially Expressed lncRNAs in HSVSMCs Treated With IL1α and PDGF

**Figure 2.**
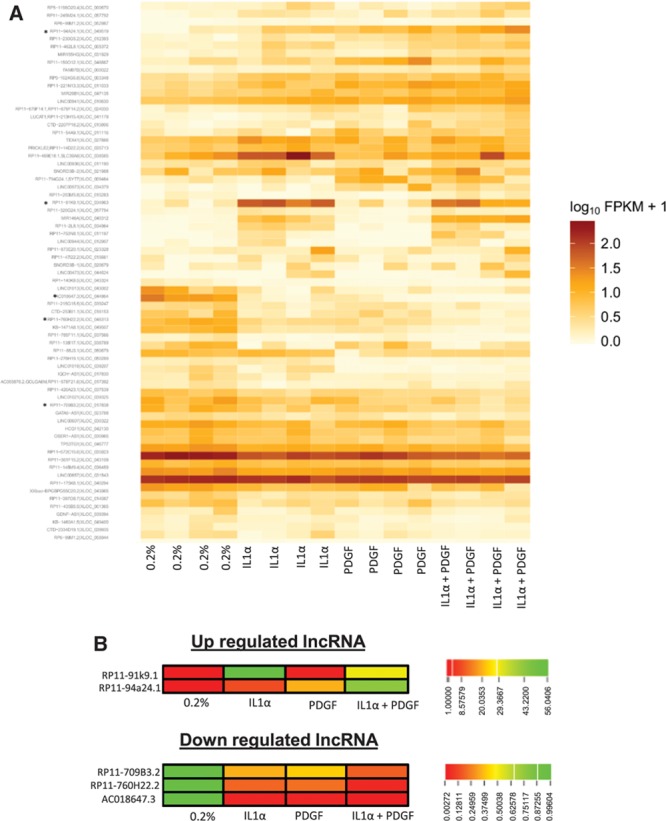
Identification of differentially expressed lncRNAs in HSVSMCs treated with IL1α and PDGF. **A**, Heatmaps showing order of differentially expressed transcripts within the 4 patient samples before and after IL1α/PDGF treatment. lncRNA selected for validation marked by *. **B**, Heatmap representing the fold change of the 5 lncRNAs selected for validation. Heatmaps represent data from RNA-seq pipeline. HSVSMC indicates human saphenous vein–derived smooth muscle cell; IL1α, interleukin-1α; lncRNA, long noncoding RNA; and PDGF, platelet-derived growth factor.

**Figure 3.**
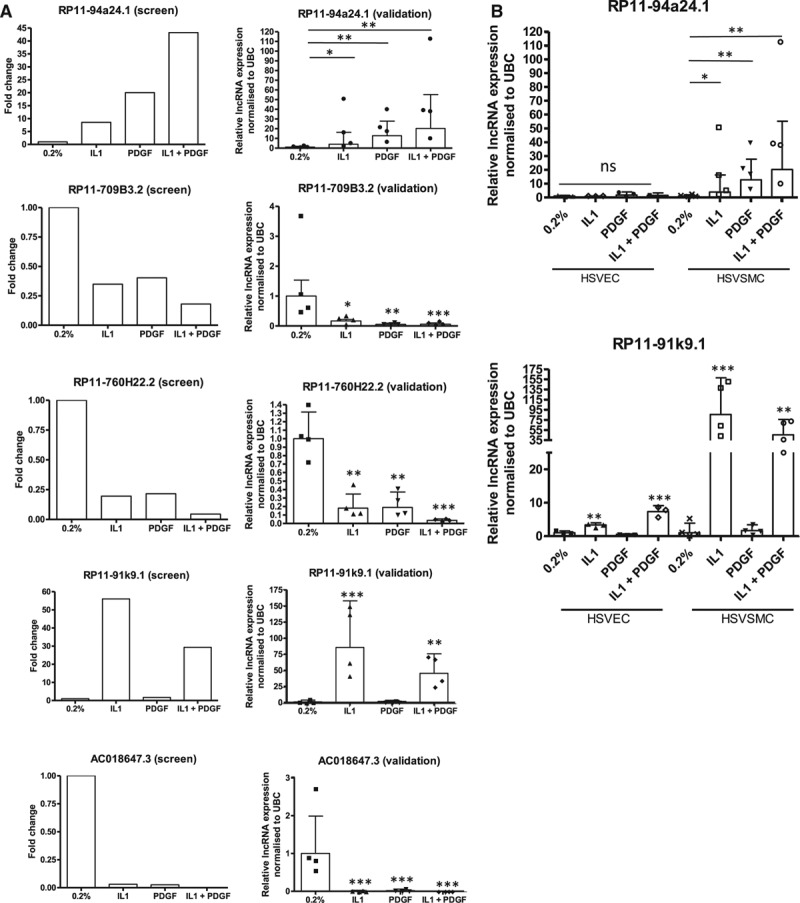
Treatment with IL1α and PDGF significantly altered lncRNA expression and showed distinct expression within vascular cell types. **A**, Graphs indicate fold change of lncRNA from RNA-seq data and subsequent validation by qRT-PCR (n=4). *P<0.05, **P<0.01, ***P<0.001 vs 0.2% condition. **B**, Basal and stimulated expression of lncRNAs 2 and 7 within HSVEC and HSVSMC (n=4 for SMC and n=3 for EC; *P<0.05, **P<0.01, ***P<0.001 vs 0.2% in each specific cell type). EC indicates endothelial cell; HSVEC, human saphenous vein–derived endothelial cell; HSVSMC, human saphenous vein–derived smooth muscle cell; IL1α, interleukin-1α; lncRNA, long noncoding RNA; PDGF, platelet-derived growth factor; qRT-PCR, quantitative real-time polymerase chain reaction; SMC, smooth muscle cell; and UBC, ubiquitin U.

We next assessed whether lncRNAs were dynamically regulated by growth factor and cytokine stimulation. Approximately 33% of reads in each condition aligned to known or predicted lncRNAs (Figure IIIA in the online-only Data Supplement). Differential expression analysis confirmed substantial differences in lncRNA expression between control and stimulated cells. Using the stringent criteria FDR ≤0.01 and log2 fold change ≥2, to declare significance and FPKM >1, to confirm quantifiable expression we identified 224, 215, and 369 differentially expressed lncRNAs following IL1α, PDGF, or dual stimulation, respectively (Figure IIIA in the online-only Data Supplement). Because lncRNAs can typically contain multiple splice variants, the numbers quoted refer to a single consensus gene model and therefore do not reflect the multiple transcripts of each lncRNA. To determine whether specific biotypes of lncRNA were enriched following HSVSMC stimulation, those differentially expressed were further subdivided according to biotype in the Ensembl database. LncRNA biotypes are based on their relative orientation to protein-coding genes; intervening lncRNA (lincRNA), antisense, overlapping and processed transcripts. With the use of control and dual stimulation as an example, the distribution of different lncRNA biotypes was as follows: intervening (45.5%), antisense (45.3%), overlapping (1.4%), and processed transcripts (7.9%; Figure IIIB in the online-only Data Supplement). Focusing on lincRNA, the candidates (control versus IL1α and PDGF, FDR<0.01, log fold change <2, FPKM>1) were ranked according to their FPKM and level of upregulation/downregulation (Figure 2A, Figure IV in the online-only Data Supplement for heat map of all conditions). A subset of the most differentially expressed transcripts was identified and validated by qRT-PCR (RP11-91k9.1, RP11-94a24.1, RP11-709B3.2, RP11-760H22.2, and AC018647.3; Figure 2B, chromosomal locations in Table I in the online-only Data Supplement). This was consistent with the RNA-seq results, showing RP11-94a24.1 and RP11-91k9.1 upregulated 20.2±30– and 45±26.4–fold, respectively, following costimulation and lncRNAs RP11-709B3.2, RP11-760H22.2, and AC018647.3 being downregulated 16-, 28-, and 1209-fold, respectively (Figure 3A; relative quantification=0.06±0.04, 0.035±0.01, and 0.0008±0.001, respectively). The dissociation curves and gel products of each primer set are shown in Figure V in the online-only Data Supplement.

### Vascular Enriched Expression of RP11-94a24.1

The expression of each lncRNA was quantified in a range of 10 normal human tissues including specimens derived from brain, gastrointestinal, reproductive, and endocrine systems. In general, lncRNAs were expressed at relatively low levels across the tissue panel. However, we observed that RP11-94a24.1 was expressed highest in the heart, whereas RP11-91K9.1 and AC018647.3 showed preferential expression within the liver and brain, respectively. RP11-709B3.2 and RP11-760H22.2 displayed highest expression in the spleen and thyroid, respectively (Figure VIA in the online-only Data Supplement). We next examined the expression of each lncRNA in primary HSVECs, HSVSMCs, and human coronary artery SMCs. All lncRNAs had higher expression in VSMCs of either venous or arterial lineage than in endothelial cells, suggesting VSMC enrichment (Figure VIB in the online-only Data Supplement). We also assessed whether the expression of these lncRNAs could be modulated by IL1α and PDGF in HSVECs as had been found in the HSVSMCs. Notably, subsequent downregulation of RP11-709B3.2, RP11-760H22.2, and AC018647.3 was not observed in HSVECs as was the case in HSVSMCs (data not shown). Stimulation of HSVECs produced a significant 3.8±0.7– and 8.7±2.1–fold upregulation of RP11-91K9.1 following IL1α and IL1α/PDGF treatment, respectively (Figure 3B). However, stimulation had no effect on RP11-94a24.1 expression (Figure 3B), indicating selective regulation in HSVSMCs. Because of the expression of RP11-94a24.1 in HSVSMCs and its cell-specific induction in response to pathological mediators of vascular injury, we focused further studies on RP11-94a24.1. We termed this lncRNA, *smooth muscle*–*induced lncRNA enhances replication* (*SMILR*). *SMILR* expression was assessed through the use of 3 independent primer sets targeting differential exons of the lncRNA. qRT-PCR revealed similar Ct and fold changes among the 3 sets, further confirming our previous data (Figure VII in the online-only Data Supplement). The longest open reading frame within *SMILR* is 57 amino acids. Analysis of this open reading frame using the Coding Potential Calculator (http://cpc.cbi.pku.edu.cn) did not reveal any similarity to known protein-coding sequences suggesting that this RNA has no protein-coding potential (data not shown).

### IL1α/PDGF Treatment Induces the Expression of *SMILR* in a Time-Dependent Manner

To investigate the longitudinal regulation of *SMILR*, we stimulated HSVSMCs with PDGF, IL1α, or a combination of both (1.5, 4, 24, 48, and 72 hours). We found significant upregulation of *SMILR* in response to PDGF as early as 4 hours after stimulation. By 24 hours, *SMILR* expression was increased by treatment with PDGF or IL1α, and both together, as well (Figure VIII in the online-only Data Supplement). The combination of PDGF and IL1α induced a synergistic increase in *SMILR* expression at 72 hours.

### Cellular Localization of *SMILR* in HSVSM Cells

Rapid amplification of cDNA ends^25^ was used to design specific RNA fluorescent in situ hybridization (FISH) probes. RNA FISH highlighted a *SMILR* isoform, consisting of an additional 6 bp at the 5′ end and 316 bp at the 3′ end (Figure IXA and IXB in the online-only Data Supplement). Rapid amplification of cDNA ends data are supported by the raw RNA-seq files (Figure XA through XC in the online-only Data Supplement).

**Figure 4.**
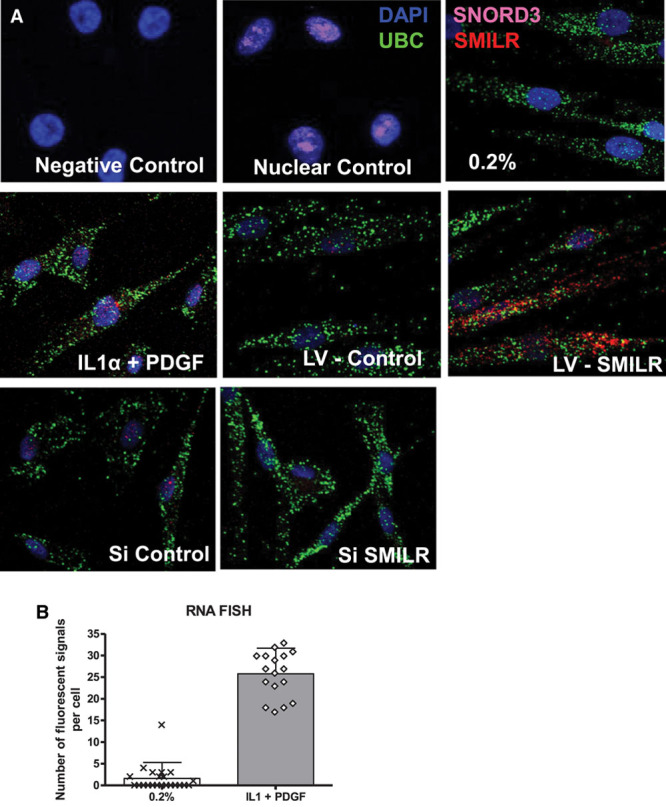
Localization of *SMILR*. **A**, RNA FISH analysis of *SMILR*, cytoplasmic *UBC* mRNA, and nuclear *SNORD3* RNA in HSVSMC. Magnification ×630 for all panels. UBC and *SNORD3* used for confirmation of cellular compartments. **B**, Quantification of lncRNA molecules per cell in indicated conditions. More than 5 images were selected at random from each condition, and at least 4 cells were counted in each image. DAPI indicates 4,6-diamidino-2-phenylindole-2-HCl; FISH, fluorescent in situ hybridization; HSVSMC, human saphenous vein–derived smooth muscle cell; IL1α, interleukin-1α; lncRNA, long noncoding RNA; LV, lentivirus; PDGF, platelet-derived growth factor; si, small interfering; UBC, ubiquitin U.

We performed RNA FISH to provide visuospatial information as to the location of *SMILR* within HSVMSCs. Negative control samples showed no fluorescent signal, whereas SNORD3 fluorescent activity confirmed the nuclear permeabilization of cells (Figure 4A). In the absence of growth factor and cytokine stimulation, HSVSMCs typically exhibited between 0 and 3 positive fluorescent signals corresponding to *SMILR* localization (Figure 4A). IL1α/PDGF treatment induced a marked increase in fluorescent signal within the nucleus and cytoplasm of HSVSMCs. Further specificity of the FISH probes was confirmed through the use of cells treated with either lentivirus containing *SMILR* or small interfering RNA (siRNA)–targeting *SMILR*. In each case, an increase and decrease in *SMILR* transcripts was observed (Figure 4A). Quantification of FISH samples is provided in Figure 4B. In the absence of stimulation, 2±3.6 *SMILR* molecules were observed. Following stimulation, 25±5 individual *SMILR* molecules were observed within the nucleus and cytoplasm (Figure 4B).

### Identifying Upstream Mediators of *SMILR* Expression in HSVSMCs

**Figure 5.**
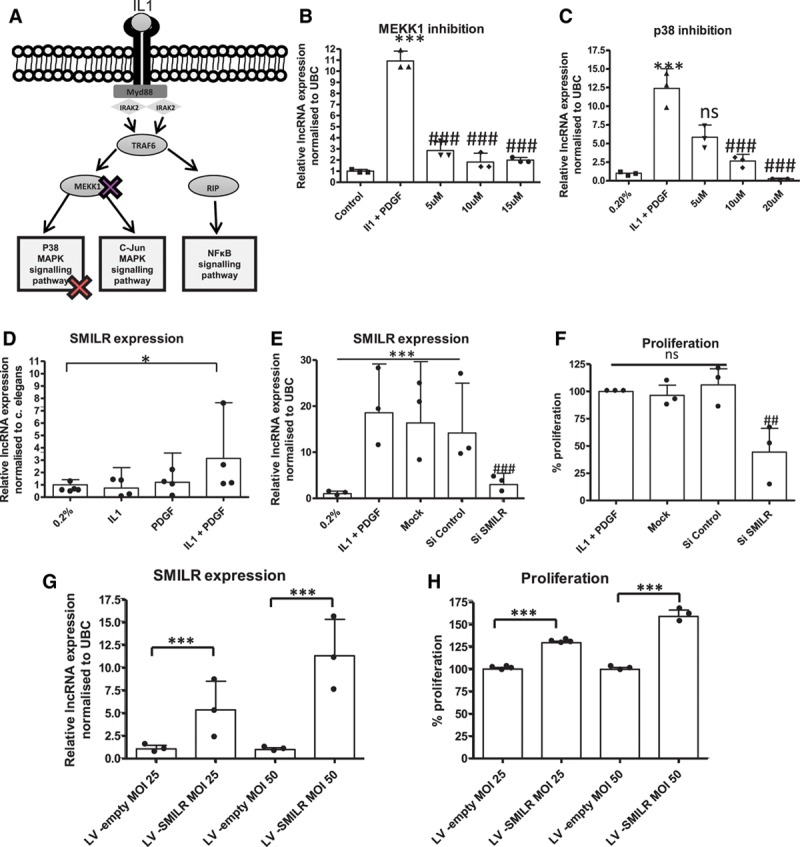
Functional regulation of *SMILR*. **A**, Schematic diagram showing specific sites of inhibition. HSVSMCs were pretreated for 60 minutes with the indicated concentration of the inhibitors. Following exposure to vehicle or 10 ng/mL IL1 or 20 nmol/L PDGF for 24 hours, expression of SMILR was determined by qRT-PCR. **B**, SMILR expression following MEKK1 inhibition. ***P<0.01 vs 0.2% media, ### *P*<0.001 vs IL1/PDGF treatment. Repeated-measures ANOVA (n=3). **C**, SMILR expression following p38 inhibition. Repeated-measures ANOVA. ***P<0.01 vs 0.2% media, ### *P*<0.001 vs IL1/PDGF treatment alone (n=3). **D**, SMILR expression in conditioned media from HSVSMCs cultured in 0.2%, IL1 or PDGF conditions. Unpaired *t* test. *P<0.05 vs 0.2% (n=4). **E**, Confirmation of the effect of siRNA targeting SMILR in HSVSMCs by using qRT-PCR (n=3). One-way ANOVA ***P<0.001 vs 0.2% control. ### *P*<0.001 vs IL1 + PDGF treatment. **F**, IL1/PDGF induced proliferation classed as 100% for analysis across patient samples, knockdown of *SMILR* inhibits EdU incorporation in HSVSMCs (n=3) One-way ANOVA vs Si control. ## *P*<0.01. **G**, qRT-PCR analysis of SMILR expression following infection with either an empty lentivirus (LV-E) or lentivirus containing *SMILR* sequence (LV-S) at an MOI of 25 (n=3) and MOI 50 (n=3) ***P<0.001 vs relevant empty control assessed via multiple-comparison ANOVA. ANOVA indicates analysis of variance; EdU, 5-ethynyl-2′-deoxyuridine; HSVSMC, human saphenous vein–derived smooth muscle cell; IL1α, interleukin-1α; lncRNA, long noncoding RNA; MAPK, mitogen-activated protein kinase; MOI, multiplicity of infection; ns, not significant; PDGF, platelet-derived growth factor; qRT-PCR, quantitative real-time polymerase chain reaction; Si, small interfering; siRNA, small interfering RNA; and UBC, ubiquitin.

It is well established that IL1α and PDGF work through distinct pathways leading to vascular cell activation. To assess the functional consequences of inhibition of these pathways on *SMILR* expression, selective pharmacological inhibitors AZD6244 (MEKK1) and SB 203580 (p38) were used (Figure 5A). Following 60 minutes of pretreatment with inhibitors, VSMCs were stimulated with IL1α/PDGF, and the expression of *SMILR* was determined at 24 hours. Pretreatment with AZD6244 (5, 10, or 15 µmol/L) prevented the induction of *SMILR* in response to PDGF and IL1α (Figure 5B), whereas inhibition of p38 with SB203580 induced a dose-dependent reduction in *SMILR* expression in response to PDGF and IL1α (Figure 5C).

### IL1α/PDGF Treatment Induces the Release of *SMILR* Into Conditioned Media

miRNAs have been reported to be secreted from cells as a means of cell-to-cell communication.^26^ To investigate whether HSVSMCs release *SMILR* as an indication of expression, we modified a method commonly used to evaluate miRNA expression.^27^ Because no endogenous control was stably expressed across all conditions in this study, an exogenous control was added to monitor extraction efficiency and to normalize data. Consequently, total RNA from *C elegans* was used as a spike-in, and *ama-1* encoding polymerase II was chosen as a control for its high constitutive expression (see Methods). Interestingly, *SMILR* was detected at low levels in media from quiesced VSMCs and those stimulated by either PDGF or IL1α, whereas conditioned media obtained from VSMC stimulated by combination contained significantly higher levels of *SMILR* (4.8±4.5–fold; Figure 5D), consistent with the increased intracellular expression of *SMILR* following costimulation of VSMC. Thus, treatment with PDGF and IL1α increased intracellular and released levels of *SMILR*.

In addition, we sought to identify if *SMILR* was encapsulated within exosomes or microvesicles. We used both ultracentrifugation, to remove cell debris, and an exosome isolation kit. Figure XIA and XIB in the online-only Data Supplement confirms the presence of microvesicles and exosomes by using Nanosight technology and the expression of the previously described miR-143 within the exosomes/microvesicles.^28^ Our data highlighted the expression of *SMILR* restricted to exosome-free media (Figure IXC in the online-only Data Supplement) and the inability to detect *SMILR* expression in the exosome/microvesicle compartment using both techniques of isolation. This observation has been confirmed by agarose gel electrophoresis (Figure XID in the online-only Data Supplement). Primer melting curves are also shown in Figure XIE in the online-only Data Supplement. Our data confirm that *SMILR* is secreted into the media and located in a nonexosome/microvesicle fraction. This could possibly be through interaction with specific membrane channels but requires additional experimentation.

In addition, we examined the release of *SMILR* following lentiviral overexpression in IL1- and PDGF-treated cells. Lentiviral overexpression resulted in a 10-fold increase in *SMILR* RNA intracellularly. However, only a marginal (not significant) increase was observed within conditioned media analyzed from infected cells (Figure XIF in the online-only Data Supplement). When this media was transferred onto additional quiesced cells, no change in proliferation was detected (Figure XIG in the online-only Data Supplement). This may suggest that the release of *SMILR* is under a stringent control mechanism and simply increasing *SMILR* expression via a lentiviral approach is not sufficient to induce the additional release of this lncRNA from the cells. In addition, these cells were stimulated with IL1α and PDGF, which strongly enhances *SMILR* expression in VSMC. The secretory machinery may have been saturated with the high levels of lncRNA within the cytoplasm. This has previously been demonstrated with miRNA where high levels of miR, via overexpression with miRNA mimics, saturated the exportin-5 pathway of endogenous miRNAs with fatal consequences.^29,30^

### Effect of Dicer Substrate siRNA–Mediated Knockdown of SMILR on HSVSMC Proliferation

We investigated the function of *SMILR* using dicer substrate siRNA (dsiRNA)–mediated knockdown and 5-ethynyl-2′-deoxyuridine incorporation. Forty-eight hours after stimulation, IL1α and PDGF treatment induced a 34±15% increase in VSMC proliferation in comparison with control (Figure XII in the online-only Data Supplement). dsiRNA *SMILR* caused 75±24% decrease in *SMILR* expression in comparison with dsi-control (Figure 5E). Following *SMILR* knockdown with dsiRNA, VSMC proliferation was reduced by 56±15% (Figure 5F). Results were confirmed through the use of a second dsiRNA targeting an alternative region of *SMILR* (Figure XIIIA and XIIIB in the online-only Data Supplement). No effect on the interferon pathway was observed via assessment of the response genes *OAS1* and *IRF7*, which have previously been linked to dsiRNA off-target effects^31^ (Figure XIIIC and XIIID in the online-only Data Supplement).

In addition, the effect of *SMILR* overexpression on VSMC proliferation was investigated. VSMCs were infected with *SMILR* lentivirus or empty control for 24 hours before stimulation. Infection at a multiplicity of infection of 25 and 50 produced a 5.5±3.5– and 11.4±4.7–fold increase in *SMILR* expression in comparison with the empty control, with no apparent toxicity effects (Figure 5G). Overexpression produced a dose-dependent increase of 1.3±0.3–fold and 1.66±0.5–fold in VSMC proliferation, respectively (Figure 5H), confirming the knockdown data.

### *SMILR* Expression Correlates With Other Proximal Genes

**Figure 6.**
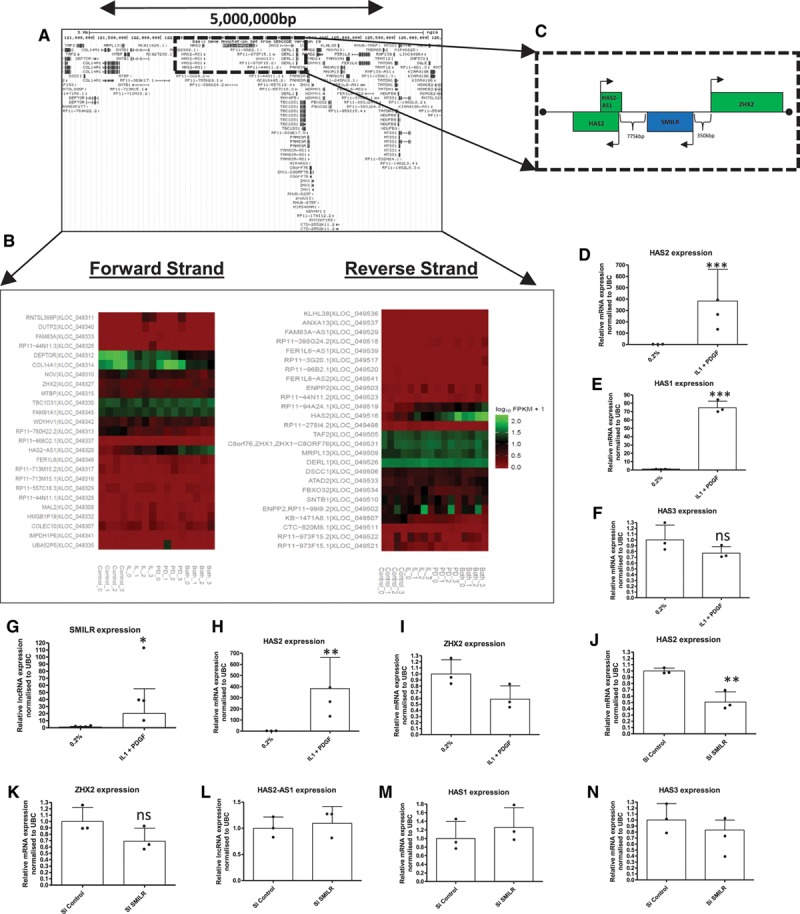
*SMILR* regulates proximal gene *HAS2* in chromosome 8. **A**, Schematic view of the 8q24.1 region showing lncRNAs and protein-coding genes over the 5 000 000-bp region from Ensemble. **B**, Regulation of protein-coding and noncoding genes within the selected region following IL1α and PDGF treatment; heatmap depicts expression of genes found with RNA-seq in 4 patient samples. **C**, Dotted line marks region containing *SMILR* lincRNA and closest protein-coding genes *HAS2* and *ZHX2*. **D**, Expression of proximal gene *HAS2* – modulated in the same manner as *SMILR* with IL1α and PDGF treatment (n=3). Unpaired *t* test: ***P<0.001 vs 0.2%. **E** and **F**, Additional HAS isoforms are differentially modulated by IL1 and PDGF (n=3). Unpaired *t* test: ***P<0.001 vs 0.2%. **G** through **I**, Validation of RNA-seq data for lncRNAs SMILR and HAS2-AS1 by qRT-PCR (n=3). *P<0.05 and **P<0.01 vs 0.2%, unpaired *t* test. **J**, Inhibition of *SMILR* expression via dsiRNA treatment significantly inhibits HAS2 expression determined by qRT-PCR **P<0.01 vs Si control. Unpaired *t* test (n=3). **K** through **N**, *SMILR* inhibition had no effect on proximal genes ZHX2 or HAS2-AS1 nor additional HAS isoforms, HAS1 or HAS3 (n=3). Unpaired *t* test. ANOVA indicates analysis of variance; dsiRNA, dicer substrate small interfering RNA; HSVSMC, human saphenous vein–derived smooth muscle cell; IL1α, interleukin-1α; lincRNA, intervening long noncoding RNA; lncRNA, long noncoding RNA; PDGF, platelet-derived growth factor; qRT-PCR, quantitative real-time polymerase chain reaction; Si, small interfering; and UBC, ubiquitin.

The expression of lincRNAs can correlate with the expression of adjacent genes and other RNAs within the genomic locale.^32^ We therefore assessed the expression of genes and noncoding RNAs within 5 million base pairs of *SMILR*, from *COL14A1* on the forward strand to *FERIL6-AS1* on the reverse strand (Figure 6A) by using the RNA-seq data set (Figure 6B). Upregulation of *SMILR* was not associated with a widespread increase in transcriptional activity within the region (Figure 6B). However, similar changes in expression in response to VSMC stimulation were observed in 2 proximal transcripts (*HAS2* and *HAS2-AS1*). *SMILR* is located ≈750 kbp downstream of *HAS2* on the same (reverse) strand and ≈350 kbp from *ZHX2* and ≈750 kbp from *HAS2-AS1* on the opposite strand of chromosome 8 (Figure 6C). The upregulation of *HAS2* was accompanied by an increase in *HAS1* but not *HAS3* following dual stimulation (Figure 6D through 6F). Interestingly, IL1α and PDGF in combination had no effect on *HAS3* expression because IL1α and PDGF have opposing effects on *HAS3* expression (full graph with single stimulation, Figure XIIIE and XIIIF in the online-only Data Supplement). In addition to *SMILR*, upregulation of *HAS2-AS1* was evident following IL1α and PDGF treatment, but not *ZHX1* in the RNA-seq data (data not shown). This observation was validated by qRT-PCR (Figure 6G through 6I).

It has been previously shown that lncRNA can modulate the expression of nearby protein-coding genes. Thus, the expression of proximal genes *HAS2*, *ZHX2*, and *HAS2-AS1* was determined following *SMILR* knockdown. RNA interference–mediated knockdown of *SMILR* significantly altered levels of *HAS2* mRNA. However, no change in the *HAS2-AS1* lncRNA or the *ZHX2* gene was observed via qRT-PCR (Figure 6J through 6L). Results were confirmed by using a second siRNA-targeting *SMILR* (Figure XIIIG through XIIII in the online-only Data Supplement). In addition, no effect on *HAS1* or *HAS3* expression was observed while *SMILR* siRNA was used, indicating that the effect of *SMILR* knockdown is specific to *HAS2* and not all isoforms of *HAS* (Figure 6M and 6N).

In addition, knockdown of *HAS2-AS1* greatly reduced *HAS2* expression with no effect on *SMILR* expression (Figure XIVA and XIVB in the online-only Data Supplement). However, the reverse experiment using *HAS2* knockdown did not affect the expression of *HAS2-AS1* or *SMILR* (Figure XIVC in the online-only Data Supplement). Finally, lentiviral-mediated overexpression did not affect *HAS1*, *HAS2*, *HAS3*, or *HAS2-AS1* expression (Figure XIVD through XIVG in the online-only Data Supplement).

### *SMILR* Expression Is Dysregulated in Unstable Human Carotid Plaques

**Figure 7.**
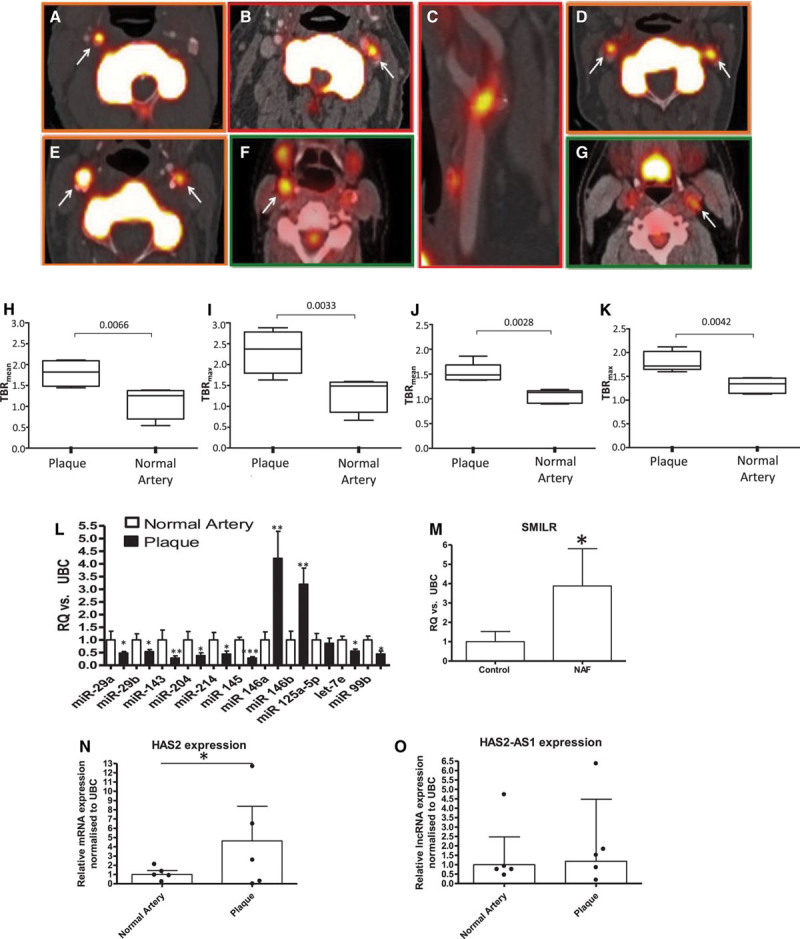
Uptake of [^18^F]fluoride and [^18^F]FDG within plaque and normal artery and changes in noncoding RNA expression within carotid plaques. Axial images demonstrating unilateral (**A**, **B**) or bilateral [^18^F]fluoride carotid uptake (**D**, **E**). **C** is a multiplanar reformat of **B**. Axial images demonstrating [^18^F]FDG carotid uptake (**F**, **G**). **H** shows the Siemens Biograph Clinical PET/CT system used for imaging. White arrows indicate carotid radioligand uptake. **H** through **K**, **L**, Uptake of tracer: MicroRNA profile of atherosclerotic plaque and paired healthy carotid controls (n=6) assessed by qRT-PCR (paired Student *t* test). Expression of *SMILR* (**M**), *HAS2* (**N**), and *HAS2-AS1*(**O**) within atherosclerotic plaque (n=5). Analyzed via qRT-PCR analysis, ***P<0.001, **P<0.01, and *P<0.05 assessed by paired Student *t* test. CT, computed tomography; [^18^F]FDG, ^18^F-fluorodeoxyglucose; PET, positron emission tomography; and qRT-PCR, quantitative real-time polymerase chain reaction.

To investigate the importance of *SMILR* in human vascular pathologies, we assessed levels of *SMILR* in unstable atherosclerotic plaques. We used 2 established inflammatory ([^18^F]fluorodeoxyglucose [FDG]) and calcification ([^18^F]fluoride) positron emission tomography radiotracers to define prospectively portions of high-risk plaque^33–35^ for RNA extraction. Plaque and relatively healthy adjacent sections of vessel were assessed from within individual patients (Table V in the online-only Data Supplement for patient characteristics). This is of key importance because it permits the assessment of noncoding RNA expression from within each micro environment (plaque versus nonplaque) from the 1 vessel. In comparison with quiescent adjacent tissue, portions of high-risk plaque showed higher uptake of both [^18^F]FDG (maximum tissue-to-background ratio 1.81±0.21 versus 1.31±1.6) and [18F]fluoride (maximum tissue-to-background ratio 2.32±0.52 versus 1.31±0.43) indicating that plaques subjected to RNA analysis had enhanced rates of inflammation (Figure 7A through 7G for image examples and Figure 7H through 7K for graphs of tracer uptake). Because noncoding RNAs have not been assessed in a similar sample set before, we first confirmed whether expression of a panel of miRNAs associated with atherosclerosis processes were dysregulated.^36^ As expected, inflammation-associated miRNAs 146a and 146b were significantly upregulated in unstable plaques in comparison with adjacent quiescent tissue, whereas miR-29 and miR-204, which are inversely associated with osteoblastogenesis and arterial calcification, were downregulated in mineralized regions of the atherosclerotic plaque.^37,38^ In addition, we also found a downregulation of the miR-143/145 cluster, which is associated with SMC differentiation and aortic aneurysm formation,^39^ an event that has previously been linked to osteogenic differentiation of SMC (Figure 7L). Thus expression of small noncoding RNAs (miRs) was associated with positron emission tomography/computed tomography-defined high-risk plaques. Therefore, we used the same cohort of samples to assess *SMILR*, *HAS2*, and *HAS2-AS1* levels. A 3.9±2.3–fold increase in *SMILR* expression was observed in high-risk plaques in comparison with adjacent stable regions of the carotid artery (Figure 7M). Intriguingly, we also observed an increased in *HAS2* (Figure 7N) but not *HAS2-AS1* (Figure 7O).

### *SMILR* Is Detectable in Human Plasma and Correlates With Inflammatory C-Reactive Protein

**Figure 8.**
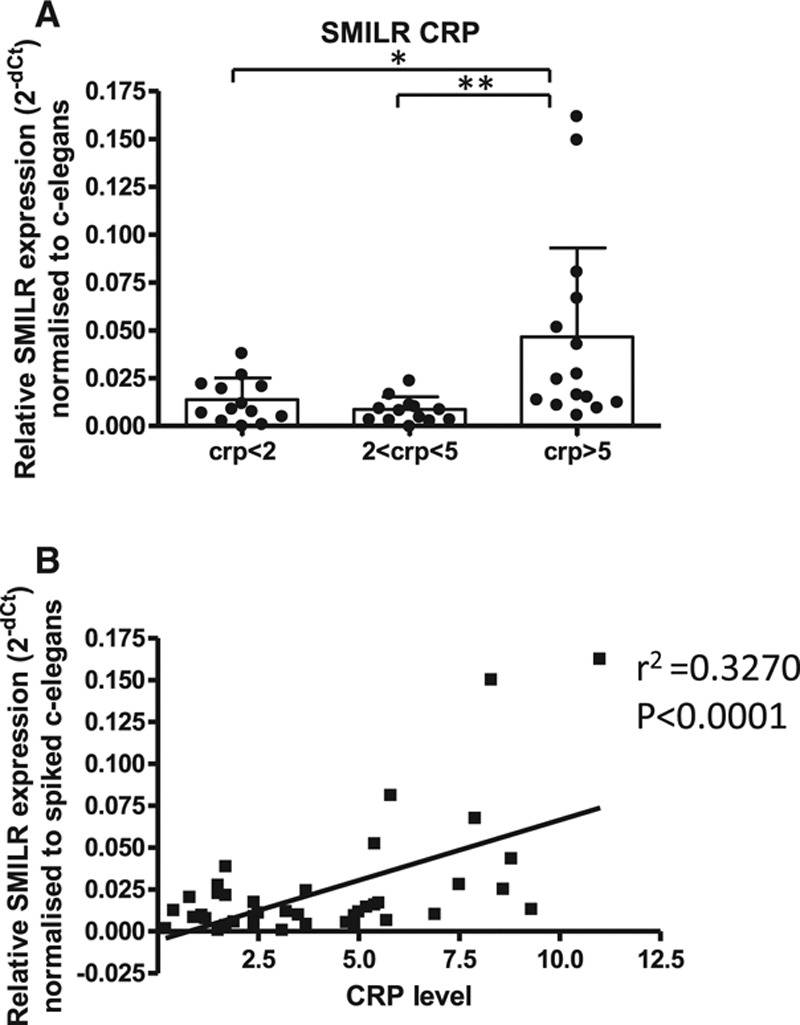
*SMILR* is detectable within plasma samples and correlates with patient CRP levels. **A**, *SMILR* expression is increased in patients with higher CRP levels (n=13 CRP<2; n=13 CRP2–5; and n=15 CRP>5; *P<0.05, **P<0.01 via 1-way ANOVA). **B**, Correlation between *SMILR* expression and CRP levels (linear regression *P*<0.0001). CRP indicates C-reactive protein; lncRNA, long noncoding RNA; and UBC, ubiquitin.

Because of the release of *SMILR* into conditioned media from VSMC following stimulation with inflammatory mediators, we evaluated whether *SMILR* was detectable in stored samples from a cohort of men with varying metabolic dysfunction. These samples were ranked in order of the serological parameter C-reactive protein (CRP) levels into 3 groups: CRP <2, CRP 2 to 5, and CRP >5 mg/L representing broad tertiles of CRP. *SMILR* showed no difference in patients with CRP levels below 2 mg/L versus 2 to 5 mg/L. However, a significant increase in *SMILR* was observed when CRP concentrations were >5 mg/ml (0.008±0.006 for CRP <2 mg/L and 0.046±0.05 for CRP >5 mg/L; Figure 8A). Furthermore, a significant positive correlation was seen between *SMILR* and CRP (*R*^2^=0.33, *P*<0.0001; Figure 8B). There was no correlation between *SMILR* and additional risk factors including age (*P*=0.66), blood pressure (*P*=0.12), BMI (*P*=0.14), or social deprivation status (*P*=0.11; Table VI in the online-only Data Supplement). Melting curves and gel products of *SMILR* primers in plasma are shown in Figure XV in the online-only Data Supplement. Further information regarding the statistical analysis of *SMILR* CRP correlation can be found in Figure XVI in the online-only Data Supplement.

## Discussion

We have shown that stimulation of HSVSMCs with PDGF and IL1α increases expression of *SMILR*. This novel lincRNA increases cell proliferation, which may be linked to its ability to regulate the proximal gene *HAS2*. In a clinical setting, we found increased expression of *SMILR* in unstable atherosclerotic plaques suggesting an association with fundamentally important vascular pathologies linked to inflammation and VSMC proliferation. We also discovered that *SMILR* can be released from cells and is detectable in plasma from patients with enhanced inflammation and thus susceptibility to atherosclerosis. These findings support the growing body of evidence that noncoding RNAs can act as mediators to modulate disease pathways.

Recent advances in RNA sequencing have demonstrated that previously thought genome deserts are in fact pervasively transcribed and are populated by lncRNAs. The use of paired-end sequencing allowed the accurate alignment of reads to the human genome (GRCh37), the 93% alignment rate met quality standards for the RNA-seq technique^40^ and ensured that our RNA-seq provided a high-quality profile of the HSVSMC transcriptome during quiescent and stimulated conditions. Notably, in comparison with control cells, protein-coding genes accounted for 3- to 4-fold greater abundance than lncRNAs. Our RNA-seq depth of 70 million reads was sufficient to identify lncRNAs within VSMC; however, it should be noted that greater read depths and the use of refined capture-sequencing technique would be beneficial to offer greater annotation of specific areas within the genome.

Analysis of the RNA-seq data pinpointed *SMILR* as an IL1α/PDGF–responsive lincRNA located on chromosome 8, 750 kbp from the closest protein-coding gene, on the same strand. This gene, *HAS2*, encodes an enzyme that synthesizes hyaluronic acid (HA), a critical component of the extracellular matrix that accumulates in human restenotic and atherosclerotic lesions.^41,42^ Our results show that knockdown of *SMILR* reduces *HAS2* expression and VSMC proliferation. This mechanism of action is supported by a number of studies demonstrating that HA can enhance VSMC proliferation and migration.^43^ Recent studies using transgenic mice with VSMC-specific overexpression of HA have found increased susceptibility to atherosclerosis^44^ and enhanced neointima formation in response to cuff injury.^45^ The ability of *SMILR* to specifically target *HAS2* with no effect on *HAS1* or *HAS3* allows a means of specifically altering *HAS2* expression, the main *HAS* isoform expressed and functioning in SMC pathology.^46^

lncRNAs can regulate other RNAs via a number of mechanisms,^47^ including changes in chromatin signatures within their locus. For example, the *HOTAIR* lncRNA is capable of repressing transcription in *trans* across 40 kbp of the *HOXD* locus.^48^ Thus *SMILR* may act as an enhancer or scaffold via interaction with the promoter region, and potentially other transcription factors of *HAS2*, to regulate expression following inflammatory cytokine stimulation. However, further detailed coimmunoprecipitation or site-directed mutagenesis studies would be required to demonstrate whether *SMILR* participates in transcription factor complexes with NF-κβ or other transcription factors. Previous work has found that *HAS2* is regulated by an additional lncRNA, *HAS2-AS1*.^49^ Interestingly, our RNA-seq data show *HAS2-AS1* expression was also upregulated by PDGF treatment alone and in combination with IL1α. However, knockdown of *SMILR* did not alter *HAS2-AS1* expression. lncRNA *HAS2-AS1* modulates chromatin structures around the gene to allow more efficient binding of the RNA polymerase 2, and enhanced *HAS2* gene expression.^49^ This suggests both *SMILR* and *HAS2-AS1* can regulate *HAS2* by independent mechanisms. Interestingly, knockdown of HAS2 did not affect either *SMILR* or *HAS2-AS1* expression, indicating that the expression of these lncRNAs is not directly linked to *HAS2* expression.

The composition of extracellular matrix assists in the determination of the stability of the atherosclerotic plaques, the phenotype of cells within it and the volume of neointima. During the progression of atherosclerosis, VSMCs are exposed to a plethora of signaling molecules, including inflammatory cytokines. Using the clinical gold-standard methods of [^18^F]FDG and [^18^F]fluoride positron emission tomography/computed tomography imaging to identify inflamed, necrotic, and mineralizing atherosclerotic plaque,^33,34^ our results indicate that miRs 29, 143, 145, 146, and 204 are differentially expressed in unstable regions of atherosclerotic plaques. These miRs have previously been linked to VSMC differentiation, inflammatory cell signaling,^50^ and VSMC calcification.^51^ The strong association and colocalization of *SMILR* with this classical miRNA profile and focal [^18^F]FDG and [^18^F]fluoride uptake within atherosclerotic plaque suggests that *SMILR* may play a role in atherosclerosis through inflammatory and proliferative pathways. In keeping with our results showing *HAS2* regulation by *SMILR*, HA content has been shown to reflect the progression of atherosclerotic disease and promotes vessel wall thickening.^52^ Indeed, HA has been implicated in the recruitment of inflammatory cells, known to play a prominent role in the initiation and progression of atherosclerotic lesion to an unstable plaque phenotype.

Our results demonstrate that *SMILR* is upregulated by a combination of PDGF and IL1α in VSMCs but not endothelial cells, suggesting that modulation of *SMILR* could specifically alter VSMC proliferation without detrimental effects on vessel reendothelialization. If this is the case, it would provide a suitable candidate to improve antiproliferative therapies because current pharmacological agents target cell proliferation in a non–cell-specific manner, events that can lead to late stent thrombosis.^53^

The ability to identify confidently a plaque, or patient, at particular risk of a major adverse cardiovascular event (ie, plaque rupture) remains an important goal of cardiovascular research. Long RNAs, both mRNA and noncoding RNA, have been previously shown to be stable in vivo for up to 3 weeks.^54^ As such, the search for prognostic biomarkers has greatly increased in recent years. *SMILR* was expressed in both the nucleus and cytoplasm of cells following stimulation and was released into the media. It will be important to determine whether the cytoplasmic copies induce functional effects, such as regulation of gene expression through posttranslational mechanisms or if they are simply being processed for release. Dual transcriptional functions of lncRNAs have been shown previously,^55^ but to date no reports of a single lncRNA affecting both transcription and translation have been published. The release of *SMILR* could affect function in neighboring cells, particularly in a vascular injury setting where phenotypic switching of VSMCs occurs in distinct areas of the vessel wall. In support of this theory, it has been shown that miR-143/145 can be transferred from VSMC into endothelial cells.^56^ This transfer produced physiological effects within endothelial cells, including modulation of angiogenesis. We also found that *SMILR* could be detected in the plasma of patients with higher CRP levels indicative of chronic low-grade inflammation. In light of our studies, we propose that this release could be attributable to the increased levels of *SMILR* in the diseased vasculature, although delineating whether plasma *SMILR* is simply a by-product of increased intracellular levels or is functionally active in disease pathology is difficult to definitely demonstrate. However, circulating levels of miR 143 and 145 are associated with in-stent restenosis and, as such, have been proposed as biomarkers.^57^ The correlation of *SMILR* and high CRP further supports its expression in low-grade chronic inflammatory settings, and proliferative settings, as well. Further large clinical cohorts will be required to ascertain if *SMILR* has prognostic potential in inflammatory vascular disease and, if so, whether it provides enhanced prognostic value over current biomarkers.

Vessel renarrowing after surgical intervention and atherosclerosis remain significant clinical problems, and HA/*HAS2*/*SMILR* have emerged as key components of these pathological processes. Administration of an siRNA targeting *SMILR* could be used to prevent renarrowing after surgical intervention for acute coronary syndrome. The use of siRNAs has been proven to be effective in phase I clinical trials. Davis et al^58^ recently showed a dose-dependent increase of siRNA delivered via nanoparticles and observed a reduction in the specific mRNA target. However, we must remain cautious, because early clinical trials in the setting of vein graft failure suggested that antisense oligonucleotides directed against E2F (edifoligide) were promising for the treatment of vein graft failure and atherosclerosis, but the subsequent phase 3 Project of Ex Vivo Vein Graft Engineering via Transfection IV (PREVENT IV) study yielded largely disappointing results.^59^ However, these studies do demonstrate that the surgical setting of coronary artery bypass grafting provides the ideal clinical setting to evaluate the clinical efficacy of these targets by gene therapy, given that the vein can be transduced ex vivo at the time of surgery. Administration of siRNA directly to the vessel would obviate the need to administer siRNA systemically and thus reduce the possibility of off-target effects. Unfortunately, there are no clear homologues of *SMILR* in the mouse. It would, however, be important to determine whether other preclinical models of human vascular disease contain *SMILR* homologues, once this information becomes available.

Taken together, these observations broaden our awareness of the complex interplay between lncRNA and protein-coding genes. The emergence of lncRNAs as regulators of gene expression will undoubtedly alter our understanding of the complex regulation network of pathological VSMC proliferation in vascular disease and may provide a means to specifically target VSMC or identify patients at risk of major adverse vascular outcomes.

## Acknowledgments

We thank N. Britton and G. Aitchison for technical assistance.

## Sources of Funding

This work is supported by the British Heart Foundation (Program grant: RG/09/005/27915 and FS11/12/28673). Dr Ballantyne is supported by the British Heart Foundation PhD Studentship (FS/12/66/30003) and Dr Baker is supported by the British Heart Foundation Chair of Translational Cardiovascular Sciences (CH/11/2/28733). Clinical PET/CT studies and Dr Vesey were funded by the British Heart Foundation (PG/12/8/29371). Drs Dweck and Newby are supported by the British Heart Foundation (FS/14/78/31020 and CH/09/002). Dr Newby is the recipient of a Wellcome Trust Senior Investigator Award (WT103782AIA). The Wellcome Trust Clinical Research Facility and the Clinical Research Imaging Center are supported by NHS Research Scotland (NRS) through NHS Lothian.

## Disclosures

None.

## Supplementary Material

## CLINICAL PERSPECTIVE

Long noncoding RNAs (lncRNAs) are a relatively new class of discovered RNA molecules that possess important regulatory functions. The rapidly expanding catalogue of lncRNAs holds promise that, in the near future, lncRNAs might become relevant to vascular disease clinically as possible biomarkers of cardiovascular events and for targeted treatment of disease. Our work indicates that dysregulation of key lncRNAs may have profound implications in regulating vascular smooth muscle cell function. In addition, we detected the release of this lncRNA in plasma samples and correlated with inflammatory C-reactive protein levels, highlighting new methods and possibilities for improved detection. The emergence of lncRNAs as regulators of gene expression and vascular function will undoubtedly alter our understanding of the complex regulation network of cell function underpinning clinical vascular disease.
