# Supplementary material for: Smooth Muscle Enriched Long Noncoding RNA (SMILR) Regulates Cell Proliferation
Source: Circulation. 2016 May 23;133(21):2050–65. doi: 10.1161/CIRCULATIONAHA.115.021019 (PMC4872641; doi:10.1161/CIRCULATIONAHA.115.021019)
Supplement: Supplementary file 1 [file cir-133-2050-s001.pdf]

SUPPLEMENTAL MATERIAL

Supplemental Methods

Tissue and Cell culture

Endothelial media supplemented 20% FCS (Life Technologies, Paisley, UK). HSVSMC media supplemented with 15% foetal bovine serum (FBS) (PAA laboratories, Yeovil, UK), 2 mM L-Glutamine (Invitrogen, Paisley, UK) 50 µg/ml penicillin (Invitrogen) and 50 µg/mL streptomycin (Invitrogen). All cells were used between passages 3-5.

HSVSMC BrdU and EdU Incorporation assay

HSVSMC proliferation was quantified using a DNA bromodeoxyuride (BrdU) incorporation assay (Millipore, Watford, UK), or EdU according to the manufacturer's instructions. Briefly, cells were plated and quiesced in 0.2% FCS media for 48 h prior to stimulation. Cells were stimulated with either 10 ng/mL IL1α, 20 ng/mL PDGF or a combination of both for the stated times. For BrdU experiments, 6 h after stimulation cells were incubated with BrdU and for EdU experiments EdU was added at the point of stimulation for the remaining time to allow cell proliferation. BrdU: after removing the culture medium, the cells were fixed followed by incubation with anti-BrdU antibody which binds the incorporated DNA. After adding the substrate solution, the immune complexes were detected using a plate reader set at dual wavelength of 450/550 nm, Victor (Perkin Elmer, Waltham, USA). EdU: following stimulation, cellular RNA was extracted as described earlier or fixed in 70% ethanol for EdU FACs analysis. EdU incorporation was quantified using Click-it EdU Proliferation assay with an Alexa Fluor 594 antibody according to the manufacturer's protocol (Life Technologies).

MEKK1 and P38 inhibitor studies

For inhibitor studies, HSVSMC were plated and quiesced for 48 h. One hour prior to stimulation, cells were incubated with either 10, 15 or 20 µM AZD6244 (MEKK1 inhibitor, Selleckchem, Suffolk, UK) or 5, 10 or 20 µM P38 (SB 203580). Cells were then maintained in either 0.2% media or stimulated with a combination of IL1α and PDGF for 24 h before RNA isolation.

5' and 3' RACE

5' 3' Rapid amplification of cDNA ends [1] was performed to determine the full length transcript of SMILR using the SMARTer RACE 5'/3' Kit (Clontech, Saint-Germain-en-Laye, France) according to manufacturer's instructions. Nested PCR was used to ensure only specific 5' and 3' products were detected (*PCR Primer sequence – Suppl. Table 1*). Following cloning into supplied cloning vector, products were sequenced.

Fluorescent *in situ* hybridisation

Custom RNA-FISH tiled probe sets were generated to all exons of SMILR. RNA FISH utilises "branch tree" technology. Briefly, a target specific probe set, containing 40 oligo probes, hybridises to the target mRNA as 20 oligo pairs. Each oligo pair forms a required platform for assembly of the signal amplification structure (tree) through a series of sequential hybridisation steps. Each fully assembled structure, covers a space of 40-50 nt of the target RNA, and has the capacity for 400-fold signal amplification. Therefore, a typical RNA probe set (containing 20 oligo pairs) has the capacity to generate 8,000-fold signal amplification. Due to this technology the company confirms single-molecule RNA sensitivity, thus each fluorescent signal corresponds to an individual lncRNA molecule.

Control SNORD3 and UBC were used as housekeepers to determine spatial location of SMILR (Panomics, Affymetrix, California, US). RNA-FISH was performed according to manufacturer's instructions (ViewRNA^{TM} cell FISH) with minor modifications for both cell and tissue experiments. For cellular analysis, HSVSMC ± IL1α/PDGF were grown on 16-mm coverslips to 80% confluency, washed in PBS and fixed in 4% paraformaldehyde supplemented with 1% glacial acetic acid. Following detergent QS permeabilisation and 1:6000 protease digest, coverslips were incubated with a combination of UBC and SMILR probe sets or UBC. Probe set buffer was used as a negative control and SNORD3 as confirmation of nuclear permeabilisation. Following probe hybridisation, cover slips were incubated with branched tree technology pre amplifier for 1 h and amplifier for 30 min. Cover slips were finally incubated with fluorescent probes, mounted onto glass slides using Prolong gold anti-fade with DAPI mounting medium (Life Technologies).

Image acquisition

Images acquired on a Zeiss 510 confocal system. At least 5 images were taken per condition. Parameters for acquisition and post analysis were identical for all conditions. Images were Z stacked to confirm nuclear localisation.

Dicer substrate siRNA (dsiRNA) mediated transfection

Double stranded dicer substrate siRNA targeting SMILR and Si-control were synthesised (Integrated DNA Technologies, Leuven, Belgium). The Si-control does not target any sequence in the human, mouse, or rat transcriptomes. Transient transfection was performed with Lipofectamine 2000 (Life Technologies). Cells were transfected with either 25 nM Si-SMILR or Si-Control. Six hours post transfection, cells were quiesced for 48 h and stimulated for a further 48 h with 0.2% media containing IL1α/PDGF.

Lentiviral mediated infection

Lentiviral vectors were produced by triple transient transfection of HEK293T cells with a packaging plasmid (pCMVΔ8.74), a plasmid encoding the envelope of vesicular stomatitis virus (VSVg) (pMDG) (Plasmid Factory, Bielefeld, Germany) and pLNT/SFFV-MCS plasmid employing polyethylenimine (PEI; Sigma-Aldrich, St Louis, USA) as previously described. Lentiviral titres were ascertained by TaqMan quantitative real-time PCR (qRT-PCR) using the following primer/probe sequences: forward, 5'-TGTGTGCCCGTCTGTTGTGT-3'; reverse, 5'- GAGTCCTGCGTCGAGAGAGC-3'; probe, 5'-(FAM)-CAGTGGCGCCCGAACAGGGA- (TAMRA)-3. SMILR was cloned into the pLNT/SFFV-MCS (kind gift from Adrian J. Thrasher, London, UK) plasmid using Platinum taq polymerase, according to manufacturer's instructions, to create pLNT/SFFV-MCS-SMILR. A confluent monolayer of smooth muscle cells were plated and infected with a multiplicity of infection of either 25 or 50, neither of which induced any form of toxicity in our cells. Following 24 h infection, media was changed to 0.2% for a further 48 h. Cells were then stimulated and EdU incorporation or SMILR expression investigated as above.

Detection of LncRNA in exosomes secreted from HSVSMC

SMILR expression in conditioned media utilising both ultracentrifugation and exosome isolation kits. RNA extraction of exosome free HSVSMC media was performed using a standard volume (15 mL). The conditioned media was centrifuged at 2000 g at 4°C for 10 min and then at 12000 g for 45 min to remove all cell debris. The supernatant was filtered (0.22 μm) followed by ultracentrifugation at 110 000 g, 4°C for 90 min (Optima L-80 XP ultracentrifuge Beckman coulter) to obtain microvesicles (MV) and exosomes and exosome free media compartments. Additional experiments were performed utilising the Total exosome isolation kit (Life technologies) following the manufacturer's instructions. The presence of microvesicles and exosomes was verified using the Nanosight technology

For exosomes and microvesicles, 700 µL of Qiazol (Qiagen) was added and 3 µL of c.elegans total RNA at 25 ng/µL and the RNA was extracted using miRNEasy mini kit (Qiagen) as previously described. For the exosome free media compartment, RNA was extracted from 2 mL and following the same protocol as describe in the manuscript. SMILR relative expression was determined in theses 2 compartments by qRT-PCR.

In Vivo Studies Atherosclerosis Studies: Patients, Imaging and Sampling

Carotid cohort

Patients with symptomatic carotid artery stenosis (≥50% by NASCET criteria [2]) scheduled to undergo carotid endarterectomy were recruited from neurovascular clinics at the Royal Infirmary of Edinburgh between January 2013 and April 2014. Exclusion criteria included a modified Rankin score of 3, insulin-dependent diabetes mellitus, women of child-bearing age not receiving contraception, severe chronic kidney disease (eGFR <30 mL/min/1.73 m^2), known iodine-based contrast media allergy, prior ipsilateral carotid intervention, prior neck irradiation, and inability to provide informed consent. Patients underwent a standard baseline clinical assessment including blood sampling (for standard clinical haematological and biochemical indices, including C reactive protein, and plasma RNA analysis) before undergoing separate [18F]-fluoride and [18F]-fluorodeoxyglucose ([18F]-FDG) positron emission tomography [3] combined with computed tomography (CT) scans with the use of a hybrid scanner (Biograph mCT, Siemens Medical Systems, Erlangen, Germany). Both of these tracers have been used by our group and others for plaque imaging and highlight high-risk actively calcifying [4] and inflamed or hypoxic atherosclerotic plaques.

For [18F]-fluoride imaging, a target dose of 250 MBq was administered intravenously. Scanning took place after a 60-min delay. Following an attenuation-correction CT scan (non-enhanced, low dose 120 kV, 50 mAs) PET imaging was performed in static mode covering 2 bed positions (15 min each) with the superior bed centered over the carotid bifurcation.

Following PET acquisition, a CT carotid angiogram was performed without moving the patient (Care Dose 4D, 120 kV, 145 mA, rotation time 0.5 s, pitch 0.8).

[18F]-FDG PET/CT was performed on a separate day. A target dose of 125 MBq was administered intravenously and scanning commenced after a 90-min delay. PET/CT acquisition was identical to [18F]-Fluoride save for a longer bed time of 20-min and a pre-scan fast of 6 h. Static images were reconstructed using the Siemens Ultra-HD algorithm (time of flight + True X) with corrections applied for attenuation, dead time, scatter, and random coincidences.

PET tracer uptake was quantified using an OsiriX workstation (OsiriX version 3.5.1 64-bit; OsiriX Imaging Software, Geneva, Switzerland). PET/CT image data were reviewed for evidence of tracer uptake, image quality and registration. The CT angiogram was examined to establish plaque presence, location and characteristics. Regions of interest [5] were then drawn on three adjacent 3-mm PET slices to incorporate the internal carotid artery plaque. Three ROI were then drawn around adjacent healthy portions of carotid artery and the lumen of the SVC to derive control values for “normal” arterial uptake and the blood pool respectively. Arterial standardized uptake values (SUV) were recorded and also indexed to blood pool activity thus giving a target-to-background-ratio (TBR).

At the time of surgery, plaques were collected immediately following excision and photographed. Two-millimeter diameter core biopsy specimens for RNA analysis were taken from regions of focally high uptake on PET and from normal tissue at the periphery of the endarterectomy specimen. These, along with the main specimen, were immediately frozen and placed in an -80°C fridge for subsequent batch analysis. Patient characteristic found in Suppl. Table. 5.

Assessment of lncRNA in human plasma

A standard volume of each plasma sample (300 µL) was used to extract RNA. Five volumes of QIAzol lysis reagent (Qiagen) was added per extraction and supplemented with spike-in RNA controls: 3.5 µL of miRNeasy Serum/Plasma Spike-In Control at 1.6 x 108 copies/µL (C. elegans miR-39 miRNA mimic; Qiagen) and 3 µL of c.elegans total RNA at 25 ng/µL. Following 5-min incubation at RT, chloroform was added at equal volumes to the starting sample. Following centrifugation (15 min; 8000 g; 4°C) the clear upper aqueous phase was used to isolate RNA as above.

Supplemental Figures

LncRNA Name	Chromosomal Location	Ensemble ID	Forward Primer sequence	Reverse Primer sequence
SMILER	chr8:123426571-123440790	RP11-94A24.1	ACCTTGGAGGTCTTGGGAGT	TTGCAGACACCTTCCAAACA
LncRNA 4	chr15:68591128-68593343	RP11-709B3.2	AAAAACTGCCACCTGTGACC	TTGGTGTAGGTCTGGGGAAG
LncRNA 6	chr8:121066919-121068440	RP11-760H22.2	CTGCATTGGAGAGACAGGAAT	AAAGCTGAAACCCTAAAGTCATTG
LncRNA 7	chr3:177534653-177617012	RP11-91K9.1	TGGCTAGGAGGGGGTCTATC	CACGGTGGCTCACACTTTTA
LncRNA 8	chr7:35756084-35774497	AC018647.3	CCAAGGTGATGAGCACAAAA	AAAGGTGGCAGAGTCCTTGA
SMILER RACE			GATTACGCCAAGCTTTGCAAACATTGGGATCAGCCGTGA	GATTACGCCAAGCTTTCTCACAGCCATGCTCTGGCCATT

Supplemental Table 1: Sybr green primer sequences. Exon spanning lncRNA primers were designed to each lncRNA to ensure no genomic DNA was assessed during qRT-PCR.

0.2% VS IL1α

Categories	Disease or Function Annotation	p-value
**Cellular Movement**	cellular movement	$1.3x10^{-36}$
**Cell Death and Survival**	necrosis	$1.6x10^{-33}$
**Cellular Growth and Proliferation**	proliferation of cells	$2.4x10^{-28}$
**Organismal Development**	angiogenesis	$2.3x10^{-26}$
**Cancer**	growth of tumour	$6.7x10^{-25}$
**Connective Tissue Disorder**	arthropathy	$8.1x10^{-24}$
**Inflammatory Disease**	chronic inflammatory disorder	$1.4x10^{-22}$
**Cellular Movement**	leukocyte migration	$1.9x10^{-23}$
**Inflammatory Response**	Inflammatory response	$4.3x10^{-23}$
**Gastrointestinal Response**	Digestive system cancer	$6.9x10^{-23}$

Supplemental Table 2: IL1α stimulation Ingenuity Pathway analysis. Top 10 disease and functional pathways predicted to be altered by IPA in HSVSM cells stimulated with IL1α.

0.2% VS PDGF

Categories	Disease or Function Annotation	p-value
**Cellular Growth and Proliferation**	proliferation of cells	$2.5x10^{-29}$
**Cell Death and Survival**	apoptosis	$3.3x10^{-25}$
**Cellular Movement**	migration of cells	$5.3x10^{-23}$
**Cardiovascular System Development**	development of the cardiovascular system	$2.3x10^{-26}$
**Organismal Development**	angiogenesis	$7.1x10^{-21}$
**Cellular Development**	proliferation of tumour cell lines	$8.5x10^{-21}$
**Cancer**	cancer	$1.5x10^{-20}$
**Cell Cycle**	mitosis	$1.7x10^{-17}$
**Cell Morphology**	morphology of cells	$5.3x10^{-16}$
**Tissue Development**	growth of connective tissue	$6.7x10^{-16}$

Supplemental Table 3: PDGF stimulation Ingenuity Pathway analysis. Top 10 disease and functional pathways predicted to be altered by IPA in HSVSM cells stimulated with PDGF.

0.2% VS IL1α + PDGF

Categories	Disease or Function Annotation	p-value
**Cellular Growth and Proliferation**	proliferation of cells	$1.0x10^{-45}$
**Cell Death and Survival**	apoptosis	$7.2x10^{-44}$
**Cancer**	cancer	$1.2x10^{-37}$
**Cellular Movement**	migration of cells	$2.3x10^{-34}$
**Gastrointestinal Response**	digestive system cancer	$7.0x10^{-30}$
**Cellular Development**	proliferation of tumour cell lines	$8.5x10^{-28}$
**Reproductive System Disease**	tumour	$4.8x10^{-27}$
**Cell Cycle**	Cell cycle progression	$6.2x10^{-26}$
**Cardiovascular System development**	morphology of cells	$2.2x10^{-24}$
**Cardiovascular System development**	angiogenesis	$4.8x10^{-24}$

Supplemental Table 4: IL1α + PDGF stimulation Ingenuity Pathway analysis. Top 10 disease and functional pathways predicted to be altered by IPA in HSVSM cells stimulated with IL1α and PDGF.

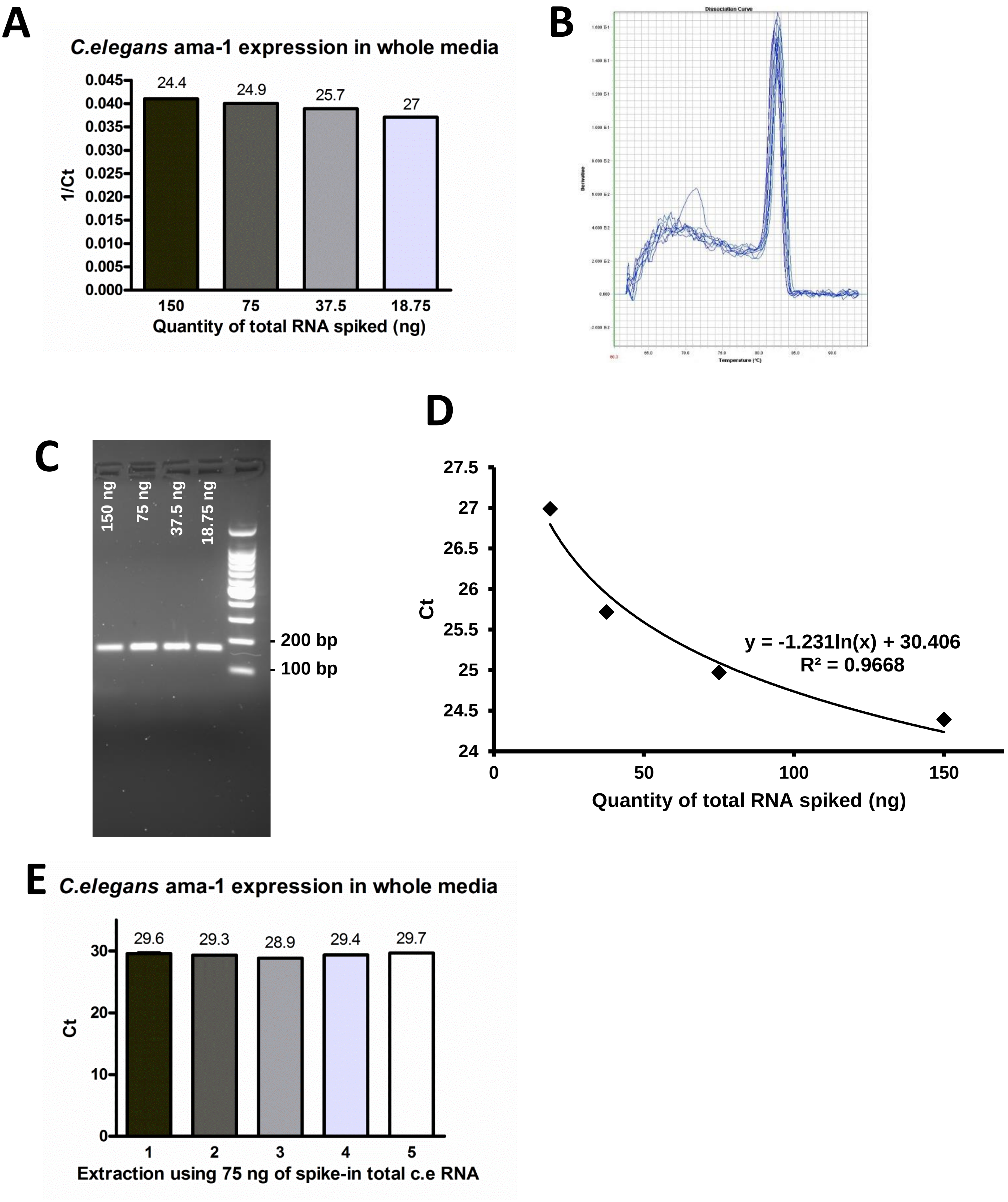

Supplemental Figure 1: Spike-in method of *C.elegans* total RNA in whole HSVSMC media. **(A):** Dose response effect of *C.elegans ama-1* expression. Expression determined by qRT-PCR and results displayed as 1/Ct. Number at the top of each histogram corresponds to Ct values. **(B):** Specificity of products analysis by melting curve. **(C):** Specificity of products analysed using agarose gel. The cDNA amplicon size has been resolved by migration on a 2% agarose gel using 100 bp ladder. **(D):** Correlation between quantity spike-in and *ama-1* expression. *C.elegans ama-1* expression follow a logarithmic function: y=-1.231ln(x)+30.406 with a coefficient of correlation r^2=0.9668. **(E):** Reproducibility of the technique. Following RNA extraction after 75 ng of spike-in total *C.elegans* RNA, *ama-1* expression was determined by qRT-PCR and the results have been displayed as Ct.

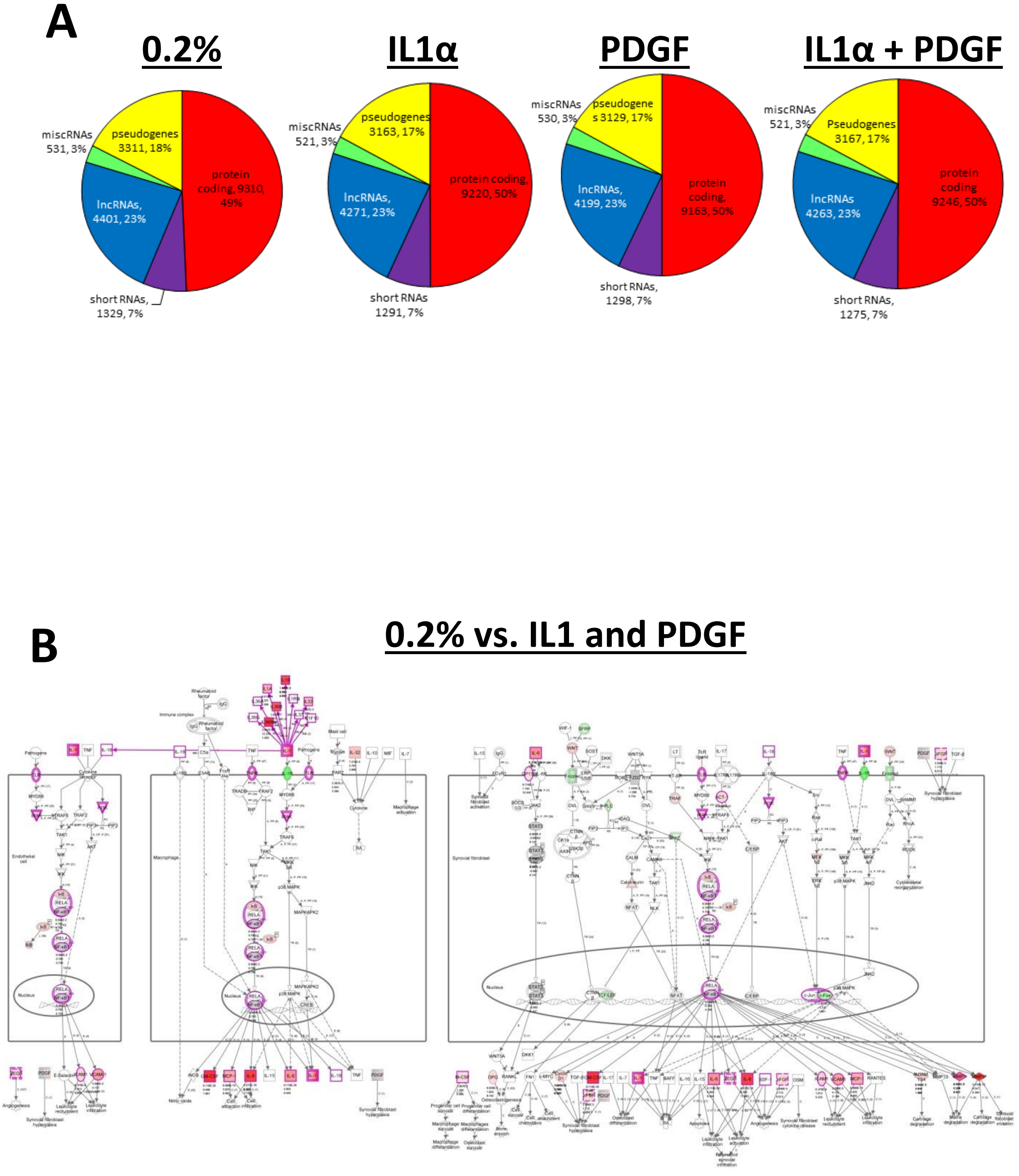

Supplemental Figure 2: **Assessment of RNA-seq data. (A):** Biotype distribution of all transcripts identified by RNA-seq analysis generated from HSVSM cells treated with IL1α and PDGF, cutoff at FPKM>0.1 **(B):** IPA analysis of top protein coding genes following IL1α and PDGF treatment.

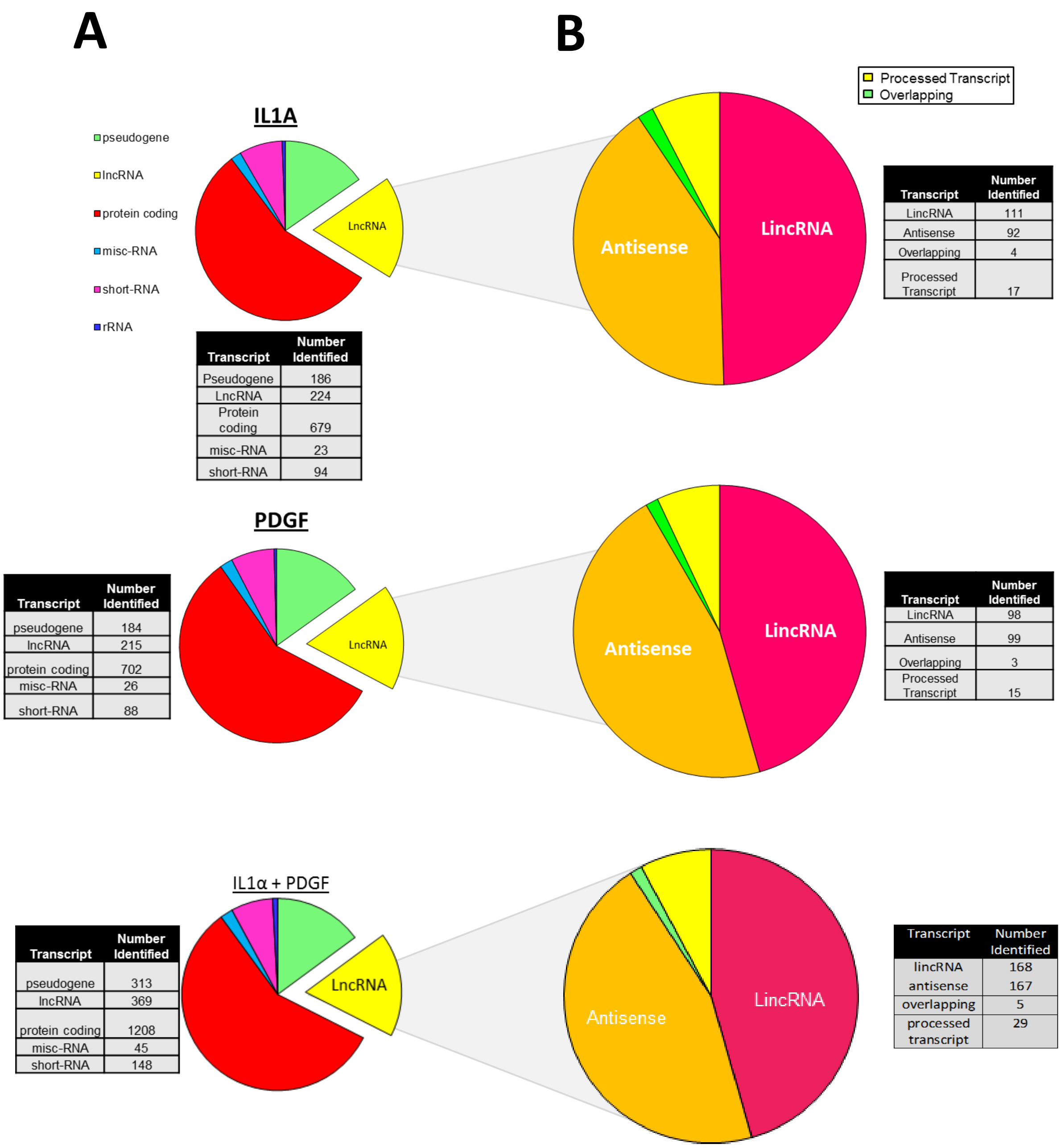

A — IL1A

Transcript	Number Identified
Pseudogene	186
LncRNA	224
Protein coding	679
misc-RNA	23
short-RNA	94

B — IL1A

Transcript	Number Identified
LincRNA	111
Antisense	92
Overlapping	4
Processed Transcript	17

A — PDGF

Transcript	Number Identified
pseudogene	184
lncRNA	215
protein coding	702
misc-RNA	26
short-RNA	88

B — PDGF

Transcript	Number Identified
LincRNA	98
Antisense	99
Overlapping	3
Processed Transcript	15

A — IL1α + PDGF

Transcript	Number Identified
pseudogene	313
lncRNA	369
protein coding	1208
misc-RNA	45
short-RNA	148

B — IL1α + PDGF

Transcript	Number Identified
lincRNA	168
antisense	167
overlapping	5
processed transcript	29

Supplemental Figure 3: **Identification of differentially expressed LncRNAs in HSVSMC treated with IL1α and PDGF.** Transcripts differentially expressed between 0.2% and stated treatment ($p<0.01$), pie chart indicates the relative percentage, and tables present numbers, of each biotype differentially expressed. LncRNAs differentially expressed between 0.2% vs IL1α/PDGF can be subdivided based on lncRNA biotype. Groups include intervening lncRNA (lincRNA), antisense, overlapping and processed transcripts.

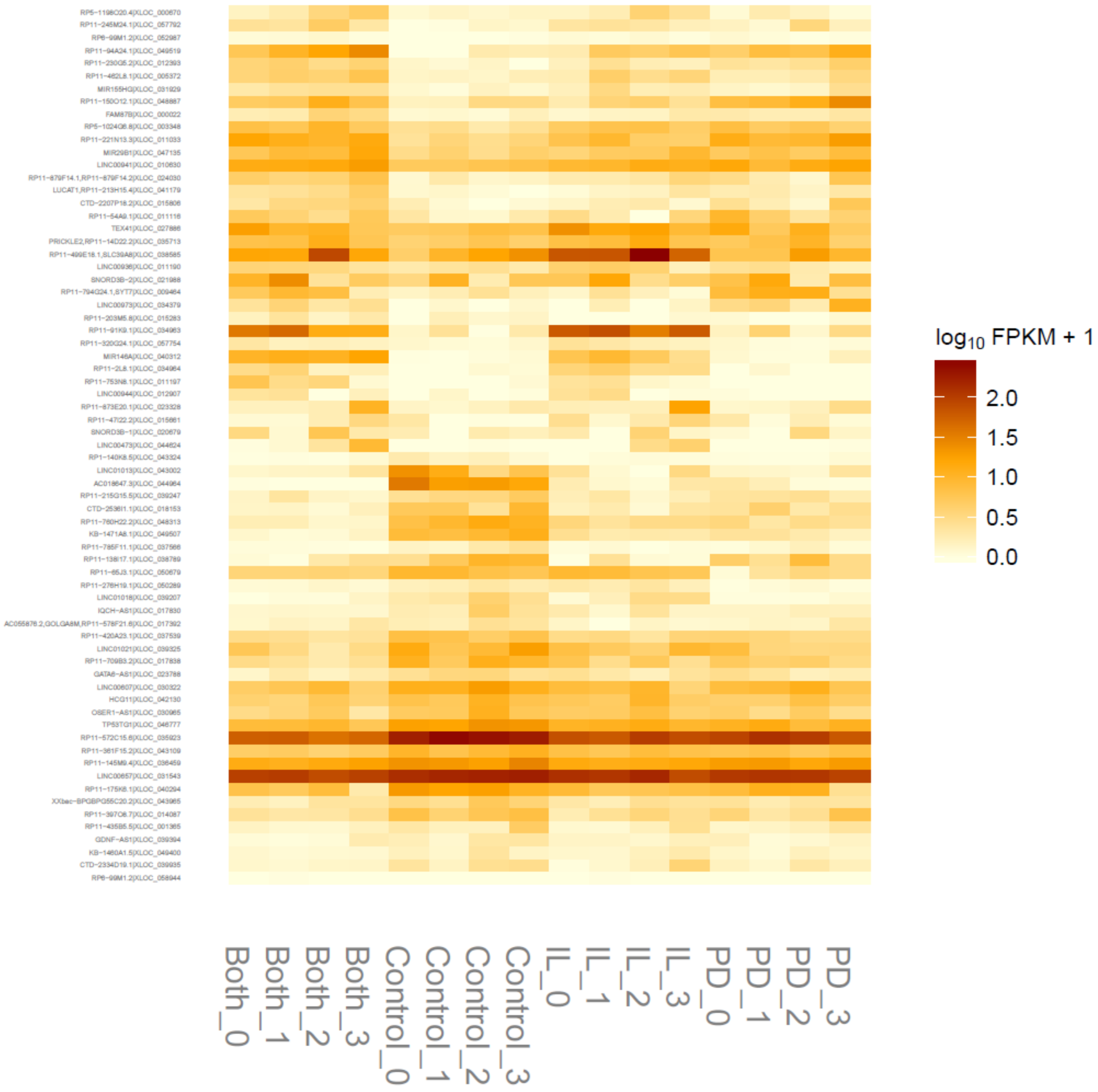

Supplemental Figure 4: **Heat map of most significantly dysregulated intervening lncRNAs across all treatment groups.** Heat map shows most significant changes in intervening lncRNA 0.2% vs IL1+PDGF treatment. LincRNA cut off using FDR<0.01, FPKM>1.

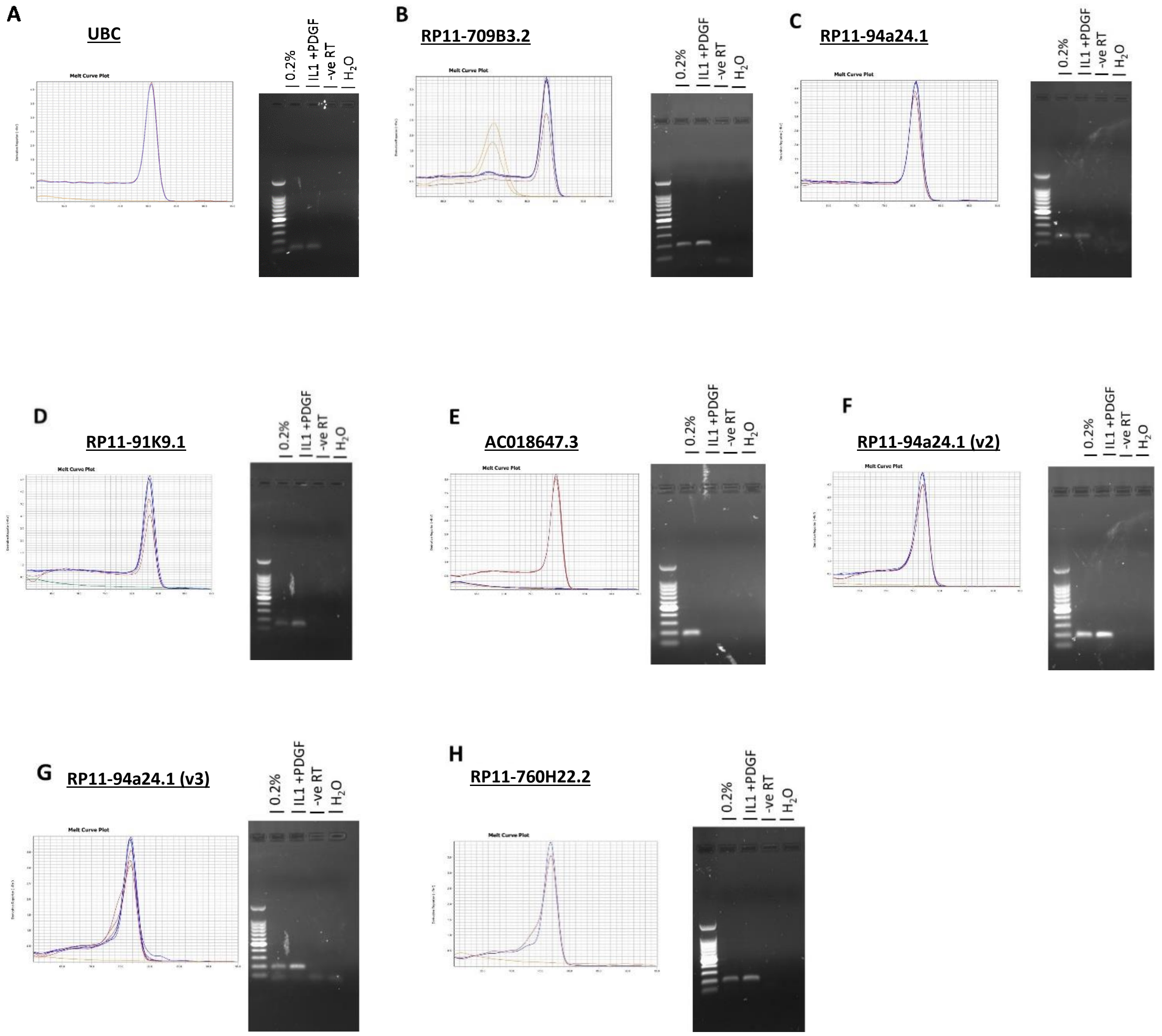

Supplementary Figure 5: Dissociation curves and gel products of PCR reactions indicating single PCR products. (A-H) Dissociation curves and gels for each lncRNA primer set. Primers were tested under 0.2% and IL1+PDGFconditions. Each gel also contains lanes containing –ve RT and H_2O samples.

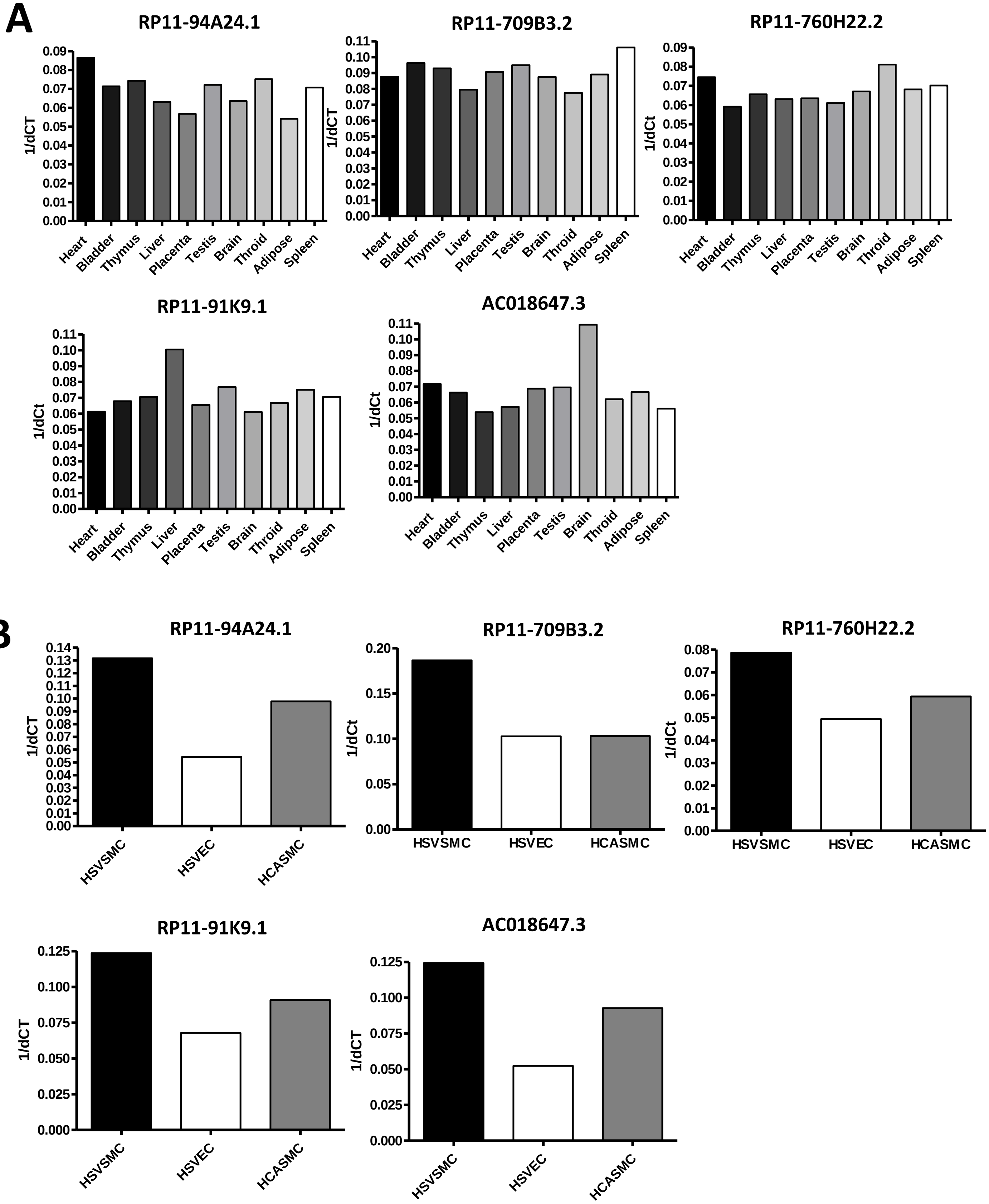

Supplemental Figure 6: LncRNA tissue and cell specificity analysis. (**A**): Expression of LncRNAs in a healthy tissue panel. Expression determined by qRT-PCR and results displayed as 1/ΔCT. (**B**): Expression of lncRNAs in unstimulated HSVSMC, HSVEC or HCAVSMC.

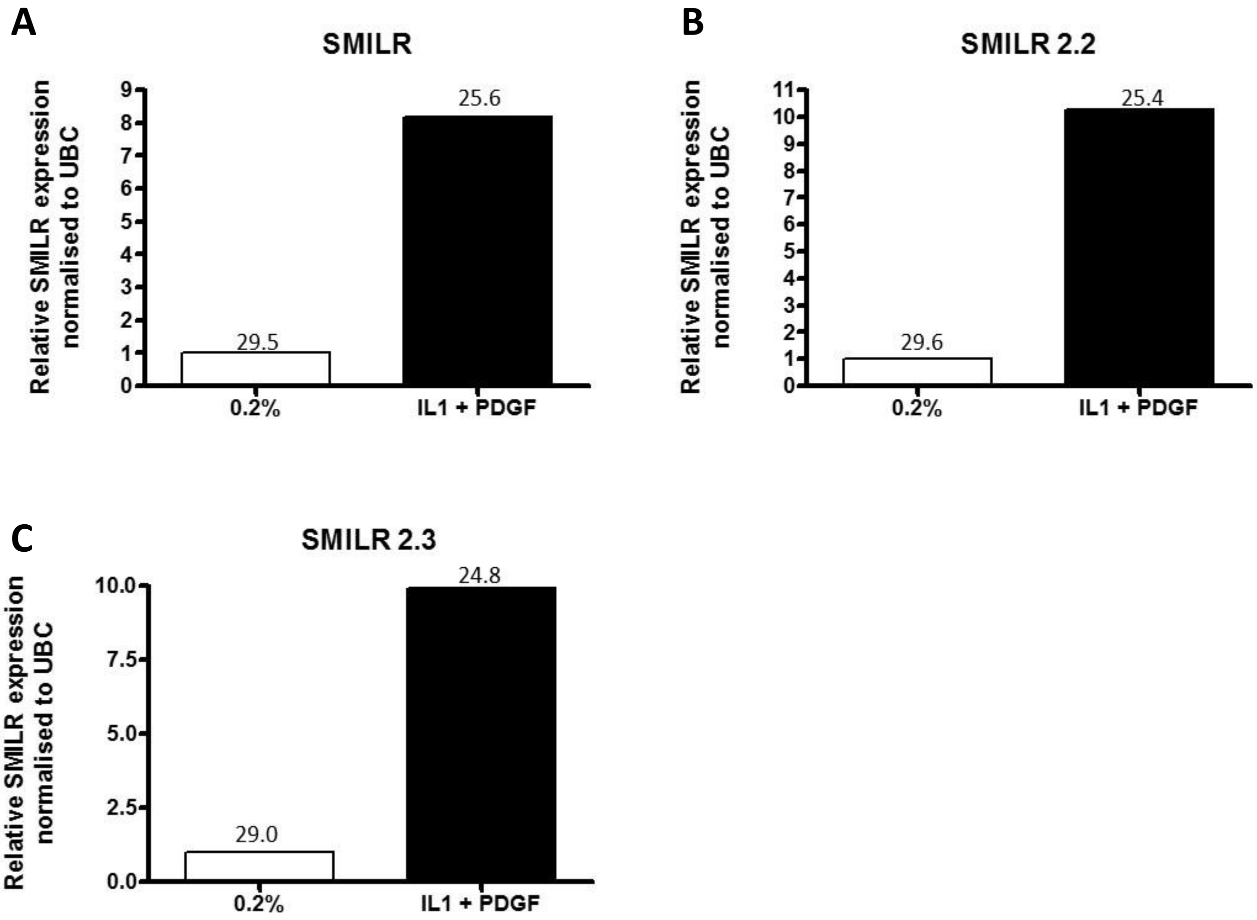

Supplemental Figure 7: Validation of additional SMILR primers. (A-C): Assessment of SMILR via qRT-PCR expression via 3 independent primer sets. The number on top of graphs represent Ct values obtained under 0.2% and dual stimulated conditions.

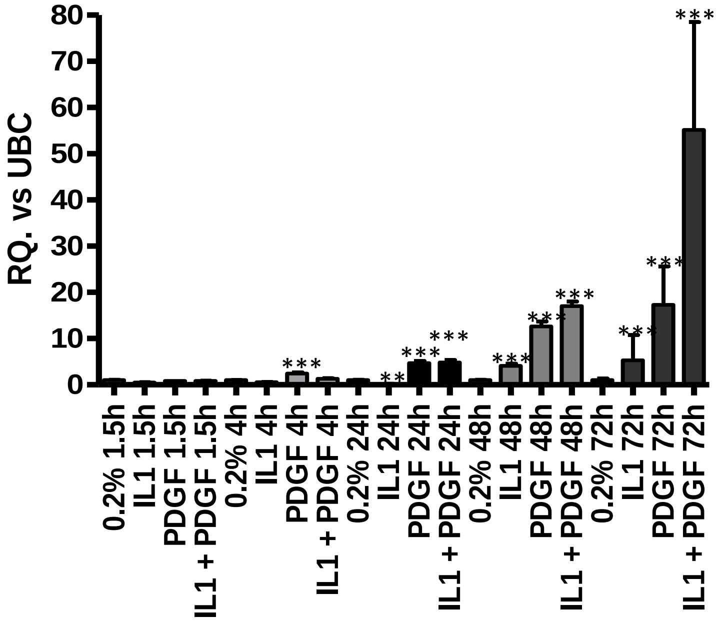

Supplemental Figure 8: **Temporal regulation of lncRNA 2 assessed by qRT-PCR.** HSVSMC were stimulated with IL1α, PDGF of a combination for the stated time points. RNA was extracted and expression determined by qRT-PCR.

A

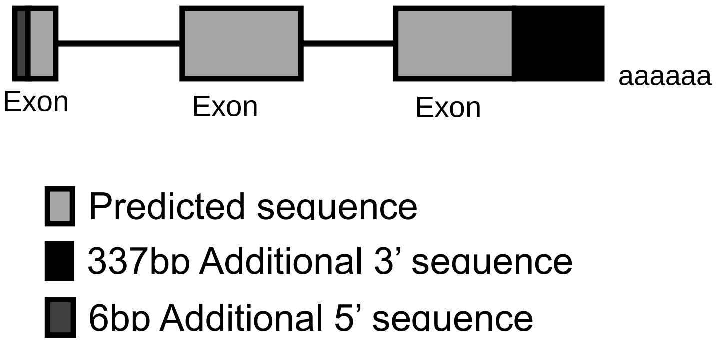

B

RP1194A24.1LncRNA2
GCTGCAAACATTGGGATCAGCCGTGACTATCCCATAACATAATATTTCTGATTTCATTCTTTT
CCTTTCTCCTACCAATTTAATCTGCAATCACTTCAAGAGAAGTCTGTTTAAAGGATATTCACA
TTCTG (intron1-11,398)
TTCACAGAGTTTGAGAGAAACTGTATTCAAGTTGCTGAAACCAAGAAGCTACACTCACGAGT
CTCACCTAAACTCGAATCTGATTTAGATGACATCATCCTGGACTTTGAGTTGATGAAACCTTG
GAGGTCTTGGGAGTAAAGCAAGTGTGATTTGCATATGATGGATATGAATTGTAATGGCCAGA
GCATGGCTGTG (intron 2- 2,269)
AGATGAAAACTCCCATTTTAGGGAACCAAGACTGAATTCCATAATTTACATGGATGTTTGGAA
GGTGTCTGCAACTTAATCTGTGTTCGTTTCTGAGATGTTGGGCAACTCCTTCTTGGAAGATG
TGGTAATGGTCTCTTCGAAAAGAAAATAATCATCTGAGTTTTGGCCAAAATAGTTGATCGGAT
TACCTATGAAAATGACTCTCACCCAACTACAAGAATGTTATGATGTAGAAACTCTAAATATAT
GAGTAATTAACTATACAACACTCCATCCCCATGTGAAAATCTTTAATCTTTTAAGATACTGAAA
TTTTGTGTATGTCTCATAATTTTCTGTATATGGTCAATGAGTTTTGCCTTAGCCATAAGTGGT
CTGTCTGAAATCTTCTCTATTATTTGTGCATTTTCTTCCTGATGTACCAAGCCTAGTCTGTTTG
TTTTTTCCTAAGAGAAAAGCAGATGATTCAACTGTGTATTTCTCAGTGTTGATATTGTGGTTT
GAGGTATTTCATAATCTTGAGTAAAATCTTGTCATAAAAAAAAAAAAAAAAAAAAA

Supplemental Figure 9: **Visual representation of full *SMILR* transcript. (A):**Grey boxes indicate the predicted *SMILR* sequence obtained from UCSC genome browser (RP11-94A24.1) . Black boxes represent additional 316 basepair sequence obtained via 3' RACE of *SMILR* transcript. ***P<0.001, ** P<0.01 and * P<0.05 vs 0.2% in each time point (1 way ANOVA). **(B):** Full length sequence of lncRNA 2.

A

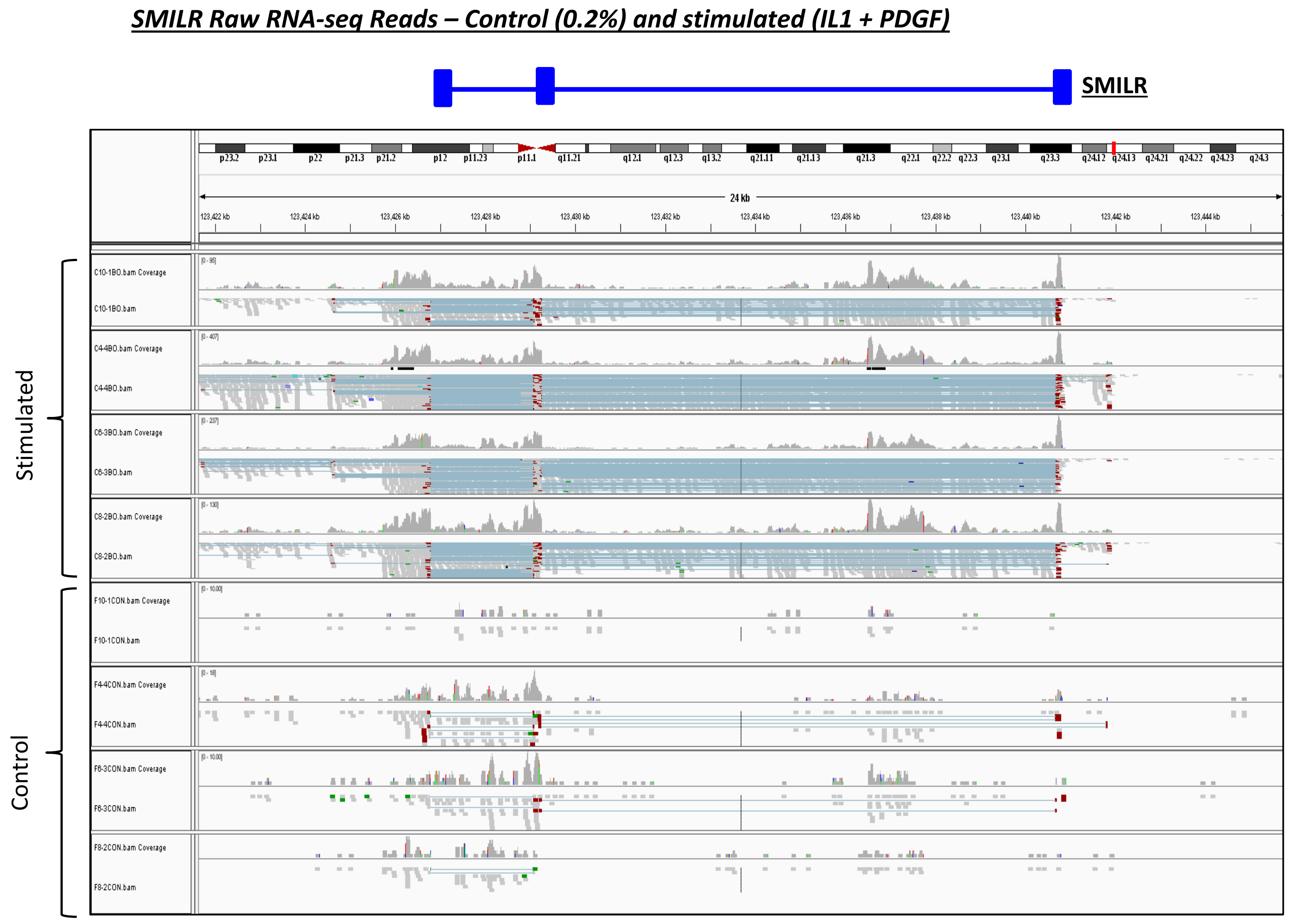

SMILR Raw RNA-seq Reads – Control (0.2%) and stimulated (IL1 + PDGF)
SMILR
24 kb
C10-1BO.bam Coverage
C10-1BO.bam
C4-4BO.bam Coverage
C4-4BO.bam
C6-3BO.bam Coverage
C6-3BO.bam
C8-2BO.bam Coverage
C8-2BO.bam
F10-1CON.bam Coverage
F10-1CON.bam
F4-4CON.bam Coverage
F4-4CON.bam
F6-3CON.bam Coverage
F6-3CON.bam
F8-2CON.bam Coverage
F8-2CON.bam
Stimulated
Control

B ***HAS2 Raw RNA-seq Reads – Control (0.2%) and stimulated (IL1 + PDGF)***

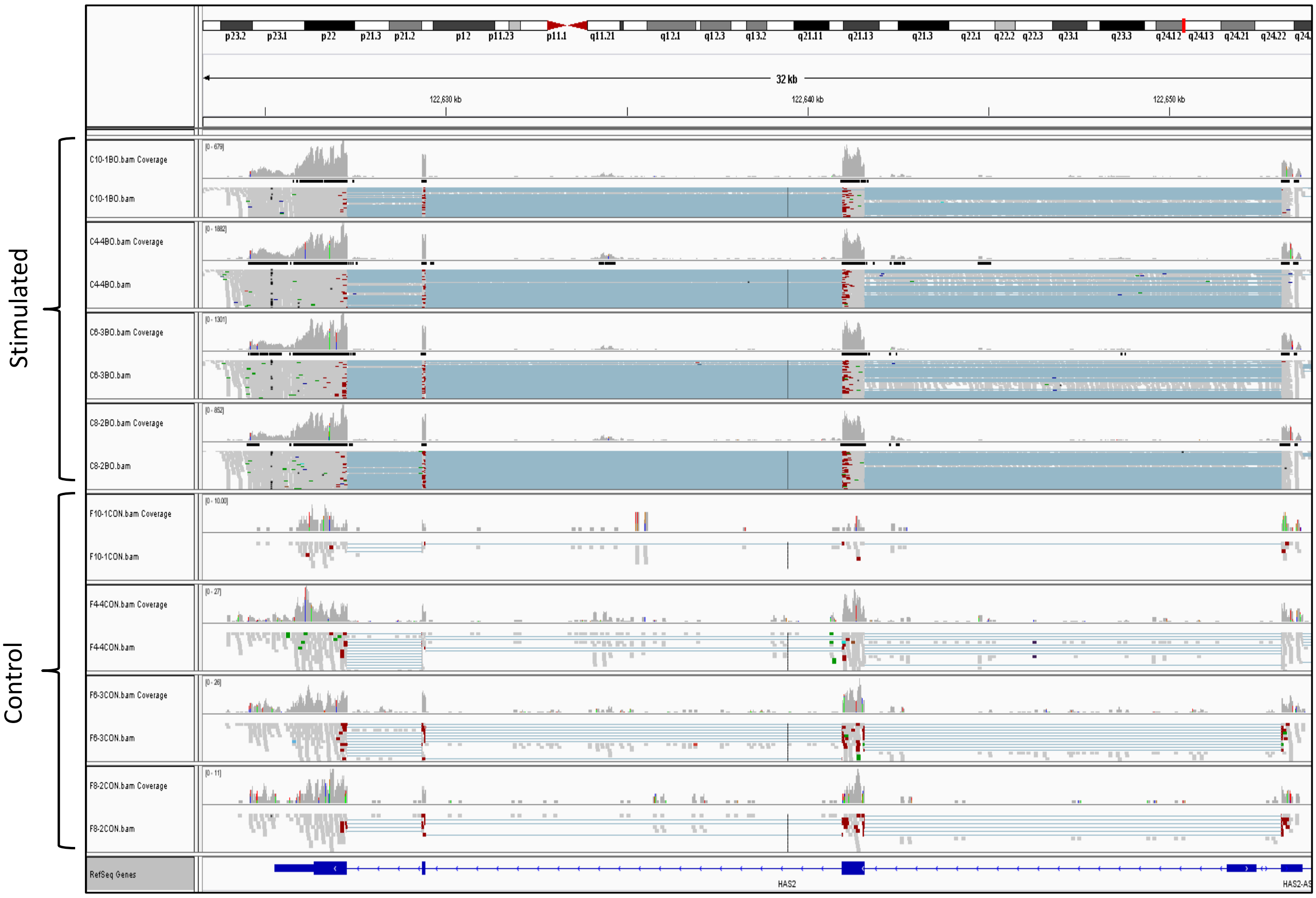

C

SMILR Raw RNA-seq Reads – Stimulated only – files expanded

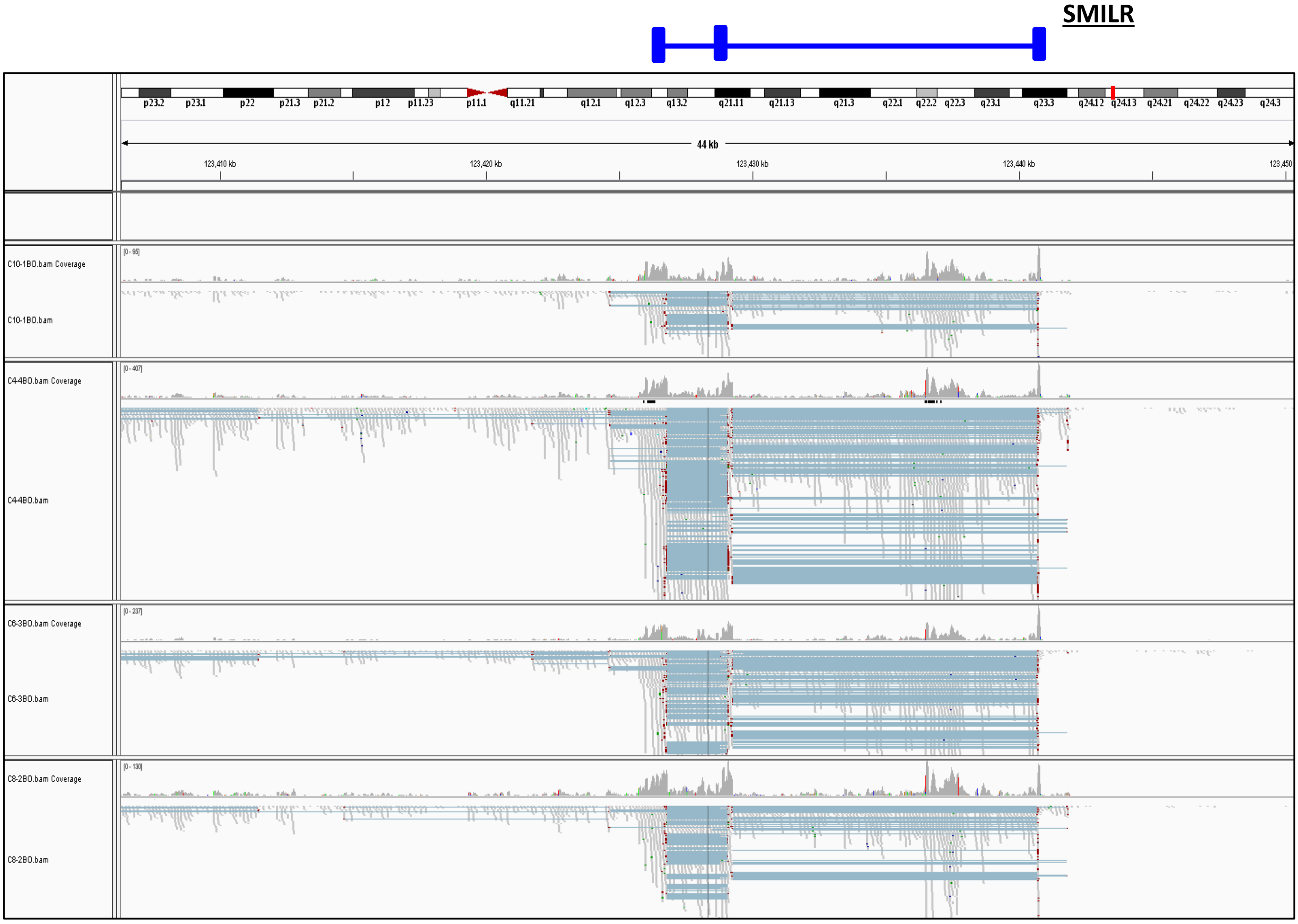

D

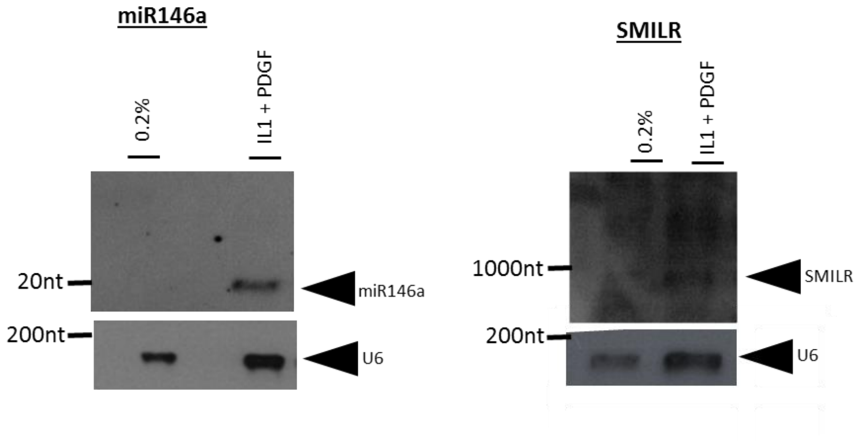

Supplemental Figure 10: Raw sequencing profiles generated utilising tophat files, constructed on integrative genome viewer (IGV). (A): Raw sequencing reads of SMILR under both basal and dual stimulated (IL1 + PDGF) conditions n=4. **(B):** Raw sequencing reads of HAS2 indicating a similar expression pattern following stimulation. **(C):** Raw sequencing reads of SMILR under stimulated conditions – expanded **(D):** Northern analysis of miR146a and SMILR RNA. U6 shown as loading control.

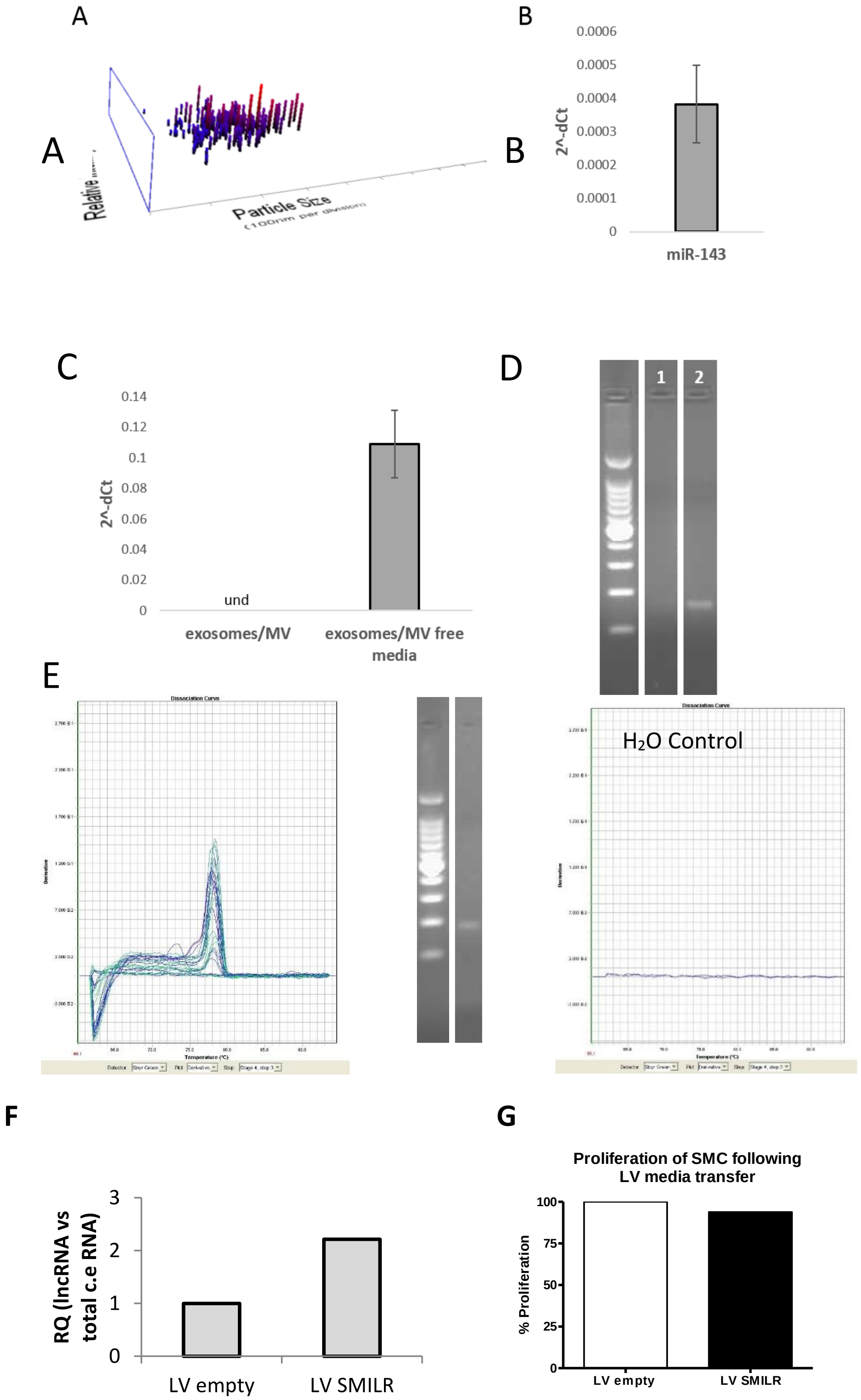

Supplemental Figure 11: Exosome isolation from HSVSMC conditioned media. (A): Size evaluation using the Nanosight of exosomes and MV isolated using the Total exosome isolation kit from 0.2% conditioned media. Sizes obtained between 70 and 600 nm. **(B):** Quantification of miR-143 in exosomes/MV isolated using the Total exosome isolation kit from 0.2% conditioned media. **(C):** SMILR expression analysed by qRT-PCR in exosomes/MV and exosomes/MV free media compartment from IL-1α + PDGF conditioned media. **(D):** Agarose gel of qRT-PCR products obtained in C; 1: exosomes/MV compartment, 2: exosomes/MV free media. **(E):** melting curves and gel electrophoresis of SMILR primer set in conditioned media**. (F):** SMILR expression from conditioned media following control lentivirus or SMILR lentivirus infection of cells. **(G):** Subsequent proliferation of quiesced cells following 48h incubation with lentivirus conditioned media.

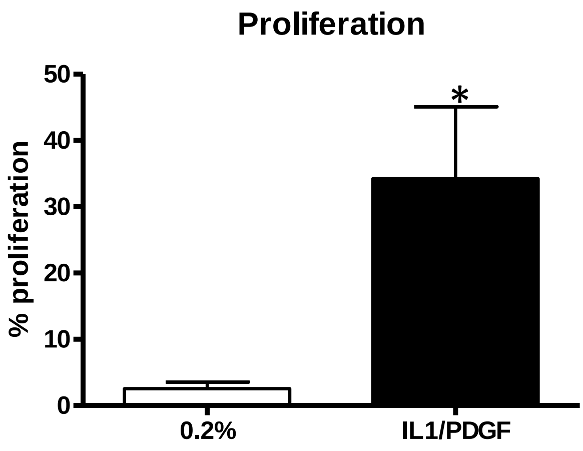

Supplemental Figure 12: Proliferation of HSVSMC 0.2% vs IL1 + PDGF treatment. P<0.05 students t test.

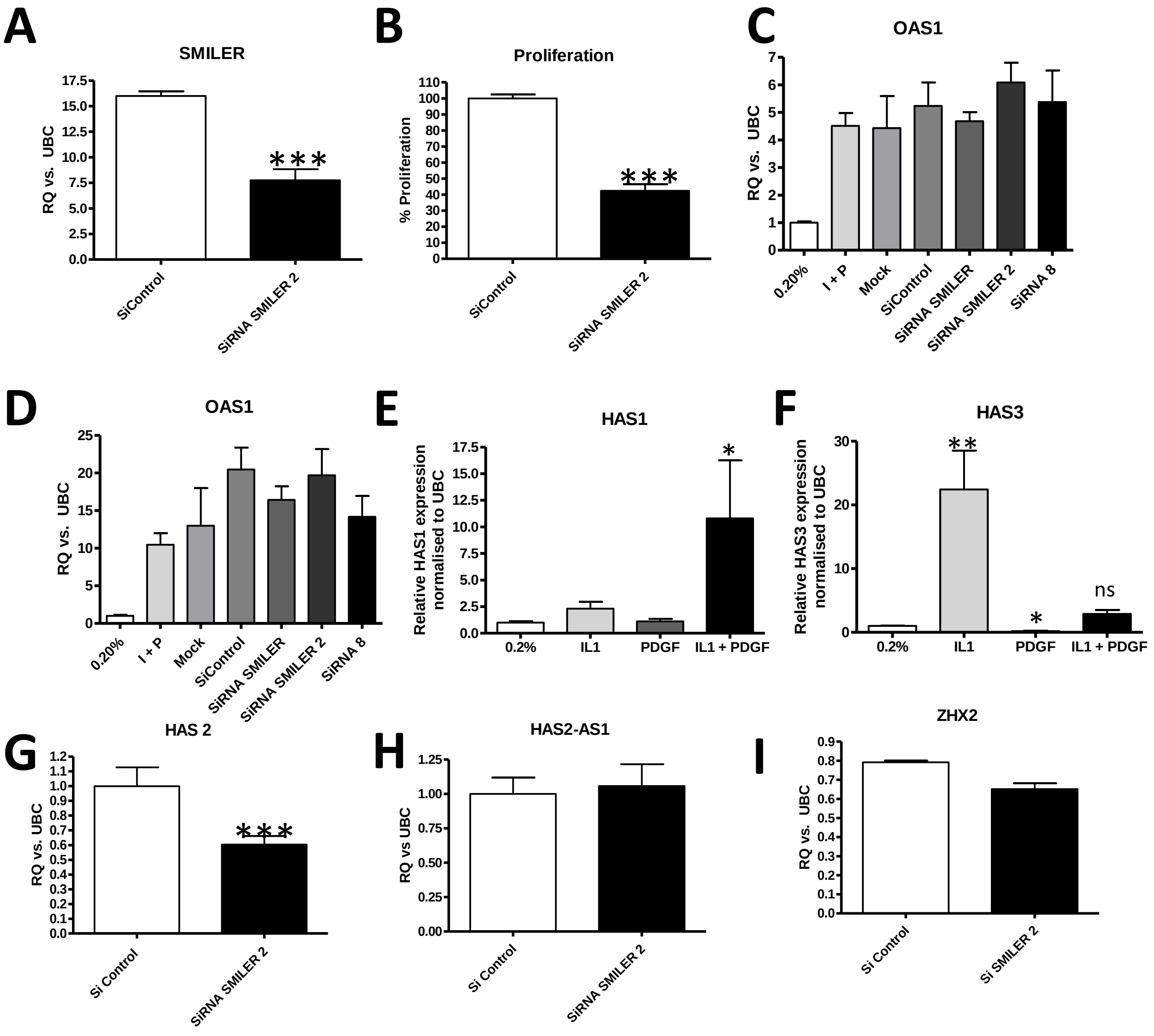

Supplemental Figure 13: (A): Confirmation of siRNA mediated down regulation of *SMILR* using second siRNA targeting a separate sequence of *SMILR*. **(B):** Confirmation of knockdown of *SMILR* using second siRNA. Analysed by students t-test ****P*<0.001 vs SiControl. **(C-D):**qRT-PCR analysis of interferon gamma associated mRNA *OAS1* and *IRF7*. **(E-F):** qRT-PCR validation of *HAS1* and *HAS3* regulation by IL1α and PDGF. One way ANOVA *P<0.05. **(G-I):** Validation of siSMILR using second siRNA targeting different section of the lncRNA.

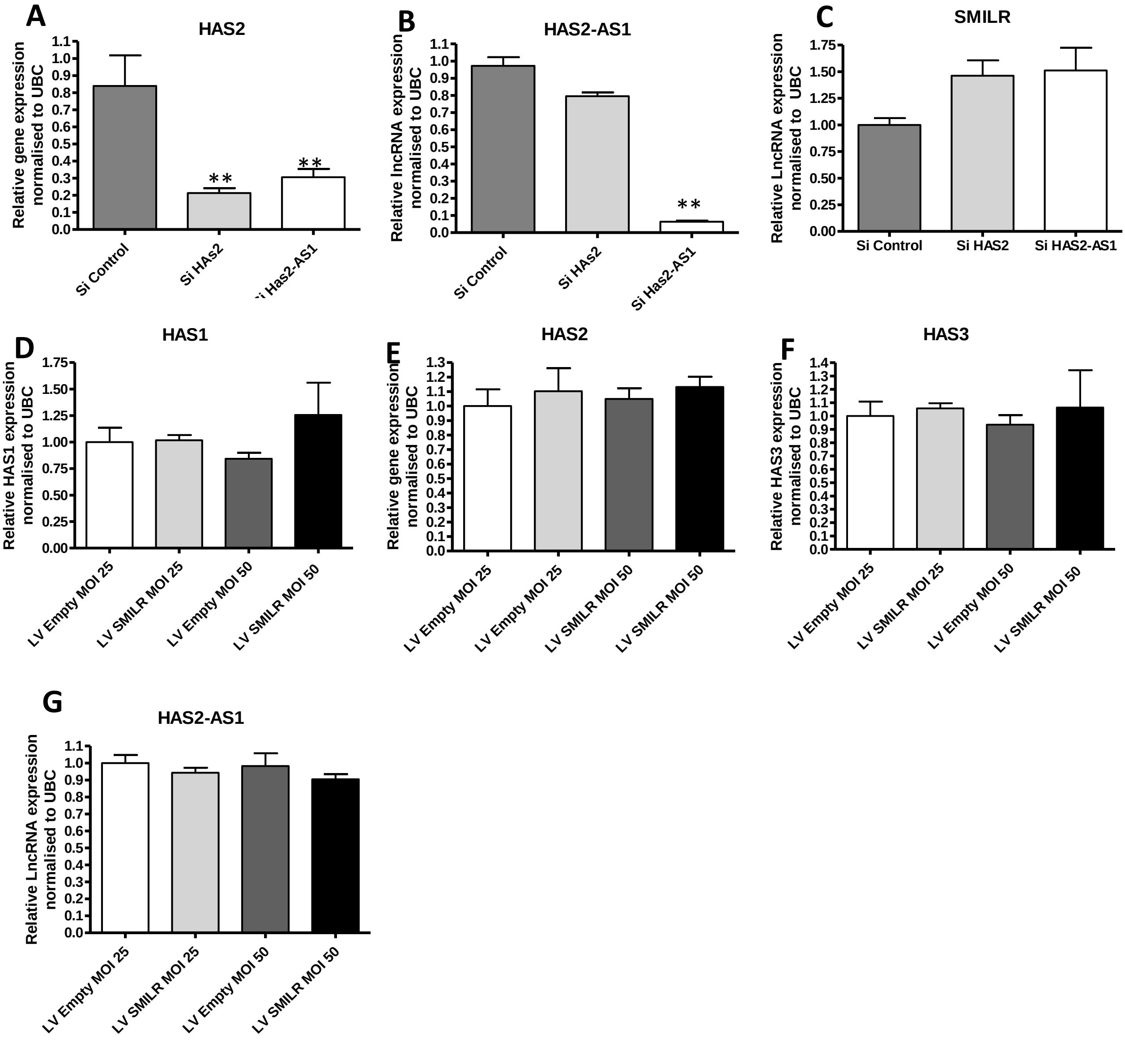

Supplemental Figure 14: Effect of HAS2 and HAS2-AS1 knockdown on SMILR expression. (A): Knocdown of HAS2 or HAS2-AS1 both reduced HAS2 expression. **(B):** Knockdown of neither HAS2 nor HAS2-AS1 affected SMILR expression levels. **(C):** Knockdown of HAS2 –AS but not HAS2 significantly reduced HAS2-AS1levels. **(D-F):** Overexpression of SMILR did not affect HAS1-HAS3 nor HAS1-AS1 expression levels.

	Carotid (n=7)
Age in years, mean (SD)	63 (13.8)
Men, n (%)	4 (57)
BMI (kg/m^2), mean (SD)	26.3 (5.8)
Systolic blood pressure (mmHg), mean (SD)	141.1 (22.5)
Diastolic blood pressure (mmHg), mean (SD)	88.4 (16.6)
Presenting syndrome, n (%)	
Stroke	2 (29)
TIA/Amaurosis fugax	5 (71)
Cardiovascular history, n (%)	
Ischemic heart disease	3 (43)
Myocardial infarction	1 (14)
Risk Factors, n (%)	
Hypertension	5 (71)
Diabetes	1 (14)
Hypercholesterolemia	7 (100)
Current smoker	3 (43)
Medication, n (%)	
Aspirin	2 (29)
Clopidogrel	5 (71)
Anti-coagulant	1 (14)
Statin	7 (100)
ACEi/ARB	3 (43)
B-blocker	2 (29)
Hematology, mean (SD)	
Hemoglobin	137.0 (23.1)
White cell count	8.1 (1.8)
Platelet count	284 (66)
Serum biochemistry, mean (SD)	
Creatinine (mmol/L)	90 (21.1)
Total cholesterol (mmol/L)	4.7 (1.3)

Supplemental Table 5- Baseline Patient Characteristics – Carotid Cohorts.

	group 1: crp<2 (n=13)	group 2: 2<crp<5 (n=13)	group 3: crp>5 (n=15)	*p* values
Age (years)	48.5 ± 1.8	48.5 ± 1.9	50.7 ± 2.1	0.66
CRP (mg/L)	1.24 ± 0.15	3.56 ± 0.28	7.09 ± 0.48	**p<0.0001**
Systolic BP (mmHg)	123 ± 2.9	131.2 ± 6.5	137.5 ± 4.6	0.12
Diastolic BP (mmHg)	77.5 ± 1.9	76.2 ± 2.0	79.0 ± 2.7	0.68
BMI (kg/m^2)	26.0 ± 0.5	28.7 ± 1.3	29.6 ± 1.7	0.14
WHR	0.96 ± 0.02	1.00 ± 0.02	0.99 ± 0.02	0.23
cIMT (mm)	0.64 ± 0.03	0.59 ± 0.03	0.64 ± 0.04	0.47
Smoking status, n (%)				0.015
Never smoker	61.5	61.5	60.0	
Ex-smoker	15.4	38.5	0.0	
Current	23.1	0.0	40.0	
SIMD quintile, n (%)				0.111
1	30.8	0.0	0.0	
2	23.1	7.7	6.7	
3	7.7	23.1	40	
4	7.7	15.4	13.3	
5	30.8	53.8	40	

Supplemental Table 6- Baseline Patient Characteristics – CRP matched plasma samples. Values are represented in mean ± SEM with *p* values calculated by one-way ANOVA or by Fisher's exact test for categorical variables.

SMILR in plasma

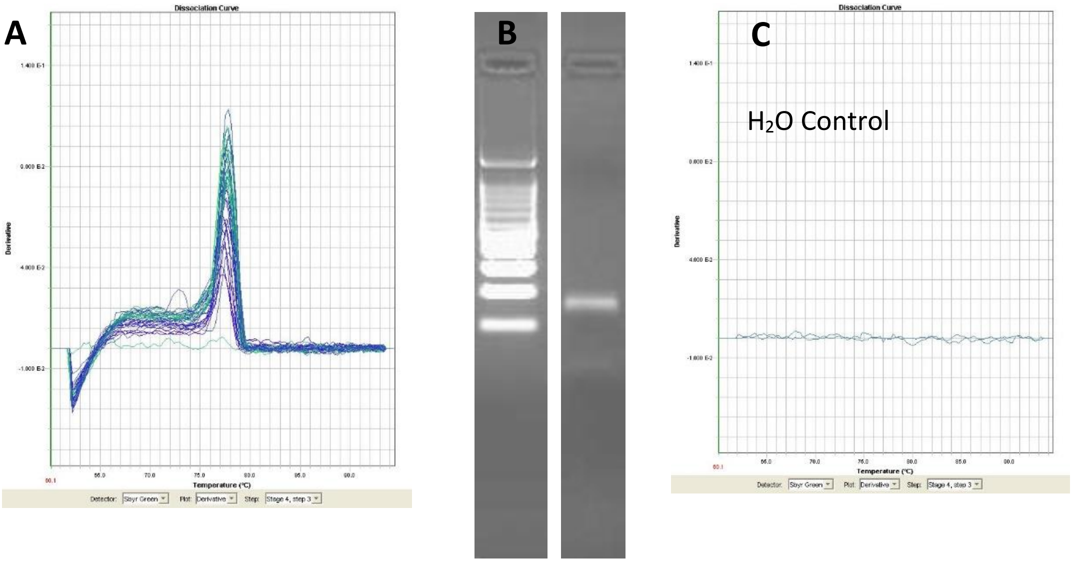

Supplemental Figure 15: Primer validation and quality control in plasma samples. (A): Melting curve for SMILR in plasma. **(B):** Agarose gel of qPCR product. **(C):** Water melting curve.

A The Pearson correlation:

		crp trans dct
Crp	1.0000	
Transdct	0.5719<br>0.0001	1.0000

So r=0.5719, r2=0.327, p<0.001

B If we take out the two outliers (the two highest dCts):

pwcorr crp transdct, sig

		crp trans dct
Crp	1.0000	
Transdct	0.389<br>0.0144	1.0000

r=0.389, r^2=0.151, p=0.014

Supplemental Figure 16: Statistical analysis of SMILR vs. CRP correlation. (A): Pearson correlation of SMILR vs. CRP utilising all data points. R=0.5719, r^2=0.327 and P<0.001. **(B):** Pearson correlation of SMILR vs. CRP omitting the 2 highest outlying points. R=0.389, r^2=0.151, p=0.014.

Supplemental References

1. Billings FTt, Balaguer JM, C Y, Wright P, Petracek MR, Byrne JG, Brown NJ, Pretorius M. Comparative effects of angiotensin receptor blockade and ace inhibition on the fibrinolytic and inflammatory responses to cardiopulmonary bypass. *Clinical pharmacology and therapeutics*. 2012;91:1065-1073
2. Hathout GM, Fink JR, El-Saden SM, Grant EG. Sonographic nascet index: A new doppler parameter for assessment of internal carotid artery stenosis. *AJNR. American journal of neuroradiology*. 2005;26:68-75
3. Upadhyay S, Ganguly K, Stoeger T, Semmler-Bhenke M, Takenaka S, Kreyling WG, Pitz M, Reitmeir P, Peters A, Eickelberg O, Wichmann HE, Schulz H. Cardiovascular and inflammatory effects of intratracheally instilled ambient dust from augsburg, germany, in spontaneously hypertensive rats (shrs). *Particle and fibre toxicology*. 2010;7:27
4. Irkle A, Vesey AT, Lewis DY, Skepper JN, Bird JL, Dweck MR, Joshi FR, Gallagher FA, Warburton EA, Bennett MR, Brindle KM, Newby DE, Rudd JH, Davenport AP. Identifying active vascular microcalcification by (18)f-sodium fluoride positron emission tomography. *Nature communications*. 2015;6:7495
5. Armand-Labit V, Meyer N, Casanova A, Bonnabau H, Platzer V, Tournier E, Sansas B, Verdun S, Thouvenot B, Hilselberger B, Doncescu A, Lamant L, Lacroix-Triki M, Favre G, Pradines A. Identification of a circulating microrna profile as a biomarker of metastatic cutaneous melanoma. *Acta dermato-venereologica*. 2016;96:29-34
